# Cholinergic midbrain afferents modulate striatal circuits and shape encoding of action strategies

**DOI:** 10.1038/s41467-020-15514-3

**Published:** 2020-04-08

**Authors:** Daniel Dautan, Icnelia Huerta-Ocampo, Nadine K. Gut, Miguel Valencia, Krishnakanth Kondabolu, Yuwoong Kim, Todor V. Gerdjikov, Juan Mena-Segovia

**Affiliations:** 1 Center for Molecular and Behavioral Neuroscience, Rutgers University, Newark, NJ USA; 2 Department of Neuroscience, Physiology and Behavior, University of Leicester, Leicester, LE1 9HN UK; 3 MRC Anatomical Neuropharmacology Unit, Department of Pharmacology, University of Oxford, Oxford, OX1 3TH UK; 4 University of Navarra, Neuroscience Program, CIMA, 31008 Pamplona, Spain; 5 IdiSNA, Navarra Institute for Health Research, 31008 Pamplona, Spain; 6 Present Address: Research line Genetic of Cognition, Italian Institute of Technology, Genova, Italy

**Keywords:** Basal ganglia, Neural circuits

## Abstract

Assimilation of novel strategies into a consolidated action repertoire is a crucial function for behavioral adaptation and cognitive flexibility. Acetylcholine in the striatum plays a pivotal role in such adaptation, and its release has been causally associated with the activity of cholinergic interneurons. Here we show that the midbrain, a previously unknown source of acetylcholine in the striatum, is a major contributor to cholinergic transmission in the striatal complex. Neurons of the pedunculopontine and laterodorsal tegmental nuclei synapse with striatal cholinergic interneurons and give rise to excitatory responses. Furthermore, they produce uniform inhibition of spiny projection neurons. Inhibition of acetylcholine release from midbrain terminals in the striatum impairs the association of contingencies and the formation of habits in an instrumental task, and mimics the effects observed following inhibition of acetylcholine release from striatal cholinergic interneurons. These results suggest the existence of two hierarchically-organized modes of cholinergic transmission in the striatum, where cholinergic interneurons are modulated by cholinergic neurons of the midbrain.

## Abstract (web summary)

Acetylcholine (ACh) in the striatum is involved in mediating action flexibility. Here the authors show that midbrain cholinergic nuclei provide a new source of Ach in the striatum, form excitatory synapses with striatal cholinergic interneurons (CIN) and contribute to instrumental learning.

## Introduction

The striatum is the main input hub of the basal ganglia. Afferents from the cortex, thalamus, and midbrain are widely distributed across its functional domains and together mediate action selection, among other functions. Acetylcholine (ACh) has a powerful influence over striatal circuits and plays key roles in adaptive behavior^1,2^. For example, reduced cholinergic transmission impairs the ability to update previous learning and enhances the possibility of interference between novel and old contingencies^3,4^.

Cholinergic markers and released ACh were considered to be exclusively associated with cholinergic interneurons (CINs), which profusely innervate the entire extent of the striatum. While they are more densely concentrated in the matrix of the dorsal striatum^5,6^, their distribution is predominantly random and heterogeneous, thus lacking functional domains^7^. Our recent work demonstrated the existence of an extrinsic source of ACh in the striatum, which originates in the pedunculopontine nucleus (PPN) and the laterodorsal tegmental nucleus (LDT) in the midbrain^8^. PPN innervation of the striatum has been shown to exist in mice, rats, and monkeys^9–13^, although its cholinergic nature was only recently revealed. In contrast to CINs innervation, cholinergic innervation arising in the midbrain is topographically organized^8^ and predominantly restricted to the anterior striatum, which receives innervation from prefrontal cortical areas^14^. At the synaptic level, PPN and LDT predominantly give rise to asymmetric specializations with dendritic shafts, suggesting excitatory connections, whereas CINs predominantly give rise to symmetric specializations with dendritic spines, suggesting inhibitory connections^8^. The evidence of two sources of ACh in the striatum, each possessing different anatomical characteristics, raises the question of whether they provide differential contributions to striatal circuits.

Cholinergic neurons of the PPN and LDT are phasically activated in response to salient events or changes in brain state^15–17^. Their activation can induce transient fast frequency oscillations in thalamic circuits, which presumably lead to cortical activation and EEG desynchronization^18^. In parallel, cholinergic neurons modulate dopamine mesolimbic circuits that innervate the striatum^19,20^, suggesting that cholinergic neurons have a converging influence on striatal circuits through mesostriatal and thalamostriatal systems^21^. The recent evidence of direct synaptic connectivity with striatal neurons^8^ further suggests that PPN and LDT modulate striatal activity. To understand the influence of the cholinergic midbrain on striatal circuits, we used a combined anatomical, electrophysiological, and behavioral approach. Our results reveal two intricately related but distinct modes of cholinergic transmission in the striatum.

## Results

### Midbrain cholinergic neurons contact striatal CINs

**Fig. 1:**
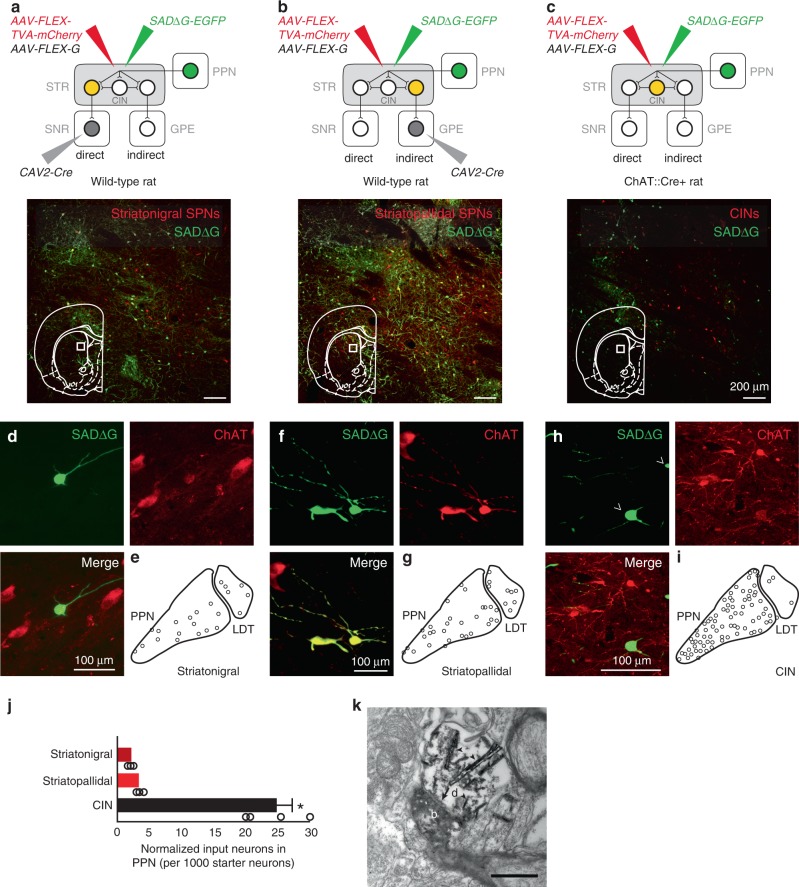
Cholinergic inputs to striatal neurons. **a** Transsynaptic labeling of striatonigral SPNs following retrograde Cre transduction in the substantia nigra pars reticulata (SNR) and pseudorabies transduction in the striatum (STR), showing both mCherry-positive neurons (red) and YFP-positive neurons (green, input neurons), overlapping red/green signals indicated starter neurons. **b** Transsynaptic labeling of striatopallidal SPNs following retrograde Cre transduction in the external globus pallidus (GPE) and pseudorabies transduction in the striatum (STR). **c** Transsynaptic labeling of cholinergic interneurons (CINs) in the ChAT::Cre rat following pseudorabies infection in STR. **d**–**i** Cholinergic input neurons **d**, **f**, **h** were observed in the pedunculopontine nucleus (PPN) and the laterodorsal tegmental nucleus (LDT) (**e**, **g**, **i**; sum of three rats). **j** Quantification of input neurons in the PPN and LDT normalized by the number of starter neurons (each circle represents one rat, obtained from *n* = 3 rats in striatonigral and striatopallidal labeling, and *n* = 4 rats in CINs [an extra animal was added to the analysis]), suggesting that CINs are preferentially targeted by PPN neurons (Kruskal–Wallis test *H*(2)=7.436, *P* = 0.0243, post hoc Tukey test: *P*_iSPNs-dSPNs_ = 0.958, *P*_iSPNs-CINs_ = 0.0001, *P*_dSPNs-CINs_ = 0.0001). **k** Electron microscope image showing an asymmetric synapse in the striatum (black arrow) formed between a cholinergic YFP + bouton (**b**; from the PPN) and a CIN dendrite **d**. Arrowheads show the accumulation of TMB crystals. Scale bar: K, 500 nm. Individual data points and mean ± SEM are shown. *P < 0.05. All experiments have been replicated at least three times.

PPN/LDT cholinergic neurons preferentially innervate dendritic shafts (76%) in the striatum with a smaller proportion contacting dendritic spines (24%) suggesting a preferential innervation of interneurons over spiny projection neurons (SPNs)^8^. To identify the postsynaptic targets of midbrain cholinergic axons, we used a monosynaptic retrograde tracing strategy to label three of the main neuronal populations in the striatum: direct pathway SPNs, indirect pathway SPNs, and CINs. SPNs were retrogradely labeled by injecting Cav2-Cre into the substantia nigra pars reticulata (SNR; Fig. 1a) or the external globus pallidus (GPE; Fig. 1b) of wild-type (WT) rats. Subsequently, two floxed viruses were co-injected into the striatum to induce the expression of a TVA receptor (AAV-FLEX-TVA-mCherry) and G-glycoprotein (AAV-FLEX-G) in SPNs. The same helper viruses were injected in the striatum of ChAT::Cre rats to target CINs (Fig. 1c). Finally, SAD∆G-eGFP was injected into the striatum of all three groups. Input neurons (eGFP-positive) were observed in the PPN and LDT of all three groups (Fig. 1d, f, h; Supplementary Fig. 1A), some of which were immunopositive for choline acetyltransferase (ChAT). The number of input neurons largely differed between the three experimental groups and revealed that CINs-injected rats gave rise to the largest number of PPN labeled neurons (Fig. 1e, g, i; Supplementary Fig. 1B). Given the marked differences in the density between SPNs and CINs in the striatum, where SPNs represent about 95% of the total striatal neurons^22^, we normalized the cell count of input neurons to the number of starter neurons (GFP+/mCherry+) in the striatum. By calculating the area of transduction (Supplementary Fig. 1C–E) and the number of starter neurons (which correlated with the expected density of each population across similar transduction areas), we found that the proportion of PPN input neurons innervating CINs is significantly larger than the proportion innervating either striatonigral or striatopallidal SPNs (Fig. 1j; Supplementary Fig. 1B; similar proportions were observed when WGA-Cre replaced Cav2-Cre, Supplementary Fig. 1F). While it is not possible to estimate the density of innervation of SPNs and CINs from quantifying the number of input neurons in the PPN, our data reveal that a larger number of PPN neurons innervate CINs compared to SPNs, thus suggesting a preferential innervation of PPN neurons to CINs over direct and indirect pathway SPNs.

Next, we used an anterograde tracing strategy based on the transduction of YFP in midbrain cholinergic axons of ChAT::Cre+ rats (*n* = 3 rats) in combination with double immunohistochemistry at the electron microscopic level. PPN YFP-positive axons were converted to a permanent peroxidase reaction using diaminobenzidine (DAB) and nickel ammonium sulfate. In addition, CIN cell bodies and processes were immunolabeled with an antibody against ChAT and revealed using tetramethylbenzidine (TMB). PPN/LDT axons, identified by the NiDAB reaction product, were observed to make synaptic contacts with dendritic processes of TMB-labeled, i.e. ChAT-positive (Fig. 1k) and unlabeled structures. Synapses formed with CIN dendrites were identified as asymmetric (Gray’s Type 1) synapses, suggesting an excitatory connection, and in line with our previous report identifying the majority of PPN-originated synaptic terminals onto dendritic shafts as asymmetric^8^. These data confirm the trans-synaptic retrograde findings and support the evidence of a direct, monosynaptic input from PPN/LDT cholinergic neurons to striatal CINs.

### Differential modulation of striatal neurons

**Fig. 2:**
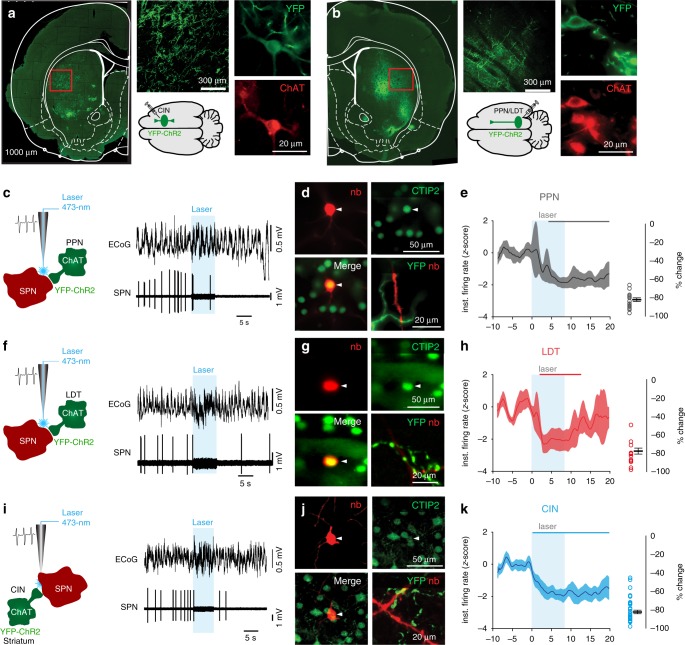
Cholinergic modulation of striatal spiny projection neurons (SPN). **a** Transduction of striatal cholinergic interneurons (CINs) in ChAT::Cre+ rats with channelrhodopsin-2 (ChR2, AAV2-DIO-EF1a-ChR2-YFP) shows dense axonal labeling in the striatum and YFP-positive somata that were immunopositive for ChAT. **b** Transduction of PPN and LDT cholinergic neurons in ChAT::Cre+ rats with ChR2 shows patches of dense axonal innervation in the striatum and YFP+/ChAT+ somata in the PPN or LDT. **c** Individual SPN neurons activity was recorded in vivo with a glass pipette during optogenetic activation (8 s, 10 Hz, 50-ms pulses) of PPN cholinergic axons. SPNs were subsequently labeled with neurobiotin (nb; *n* = 29 neurons from *n* = 12 rats). ECoG recording shows no associated change in brain state during the stimulation. **d** Only neurobiotin-labeled SPNs immunopositive for CTIP2 (COUP TF1-interacting protein-2, immunohistochemical marker for SPNs) and surrounded by YFP-positive axons were used for further analyses. **e** The normalized instantaneous firing rate of all SPNs that responded to laser stimulation of PPN cholinergic axons shows a slow inhibition during, and after, blue-light stimulation (color line in the top represents the time points during which the responses were significantly different from the baseline; cluster-based permutation test, *P* < 0.05). **f**–**h** Same experimental design to assess modulation of striatal SPNs by LDT cholinergic axons (*n* = 19 neurons from *n* = 15 rats). LDT cholinergic axon stimulation induced a reduction in the firing rate of SPNs, similar to PPN cholinergic axon stimulation. **i**–**k** Same experimental design to assess modulation of striatal SPNs by cholinergic axons arising from local CINs in the dorsal striatum (*n* = 43 neurons from *n* = 17 rats). CINs axon stimulation induced a reduction in the firing rate of SPNs, similar to the responses of the midbrain. Following cholinergic axon stimulation from each of the three origins, SPNs showed a similar reduction in the firing rate (% change in firing rates: PPN, −79.58 ± 2.56, *n* = 29; LDT, −78.39 ± 1.92, *n* = 19; CINs, −79.24 ± 2.43, *n* = 43; one-way ANOVA *F*(2,64) = 0.042, *P* = 0.959). Individual data points and mean ± SEM are shown. *P < 0.05. All experiments have been replicated at least three times.

**Fig. 3:**
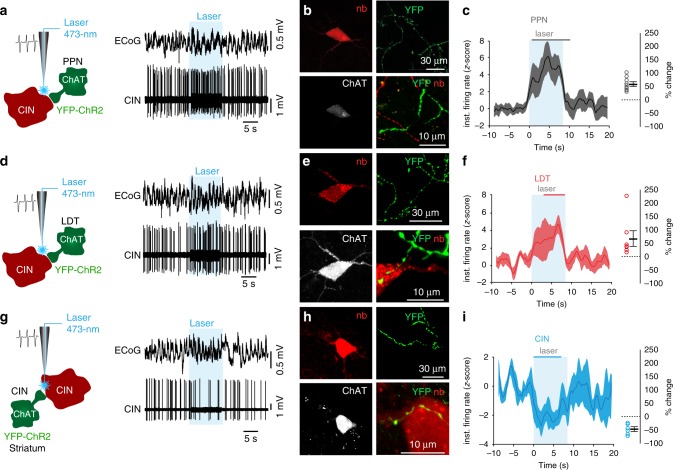
Cholinergic modulation of cholinergic interneurons (CINs). Individual CINs activity was recorded in vivo during optogenetic activation (8 s, 10 Hz, 50-ms pulses) of cholinergic axons originating in the PPN (**a**–**c**; *n* = 19 neurons), LDT (**d**–**f**; *n* = 13 neurons) or from local CINs (**g**–**i**; *n* = 10 neurons). CINs were subsequently labeled with neurobiotin. Only neurobiotin-labeled CINs that were immunopositive for ChAT and surrounded by YFP-positive axons were used for further analyses **b, e, h**. The normalized instantaneous firing rate of all CINs that responded to laser stimulation of PPN **c** and LDT **f** show similar increase in firing rate shortly after stimulation, whereas non-transduced CINs (YFP/ChR2-negative; **i**) were strongly inhibited during stimulation (color lines in the top represent the time points during which the responses were significantly different from the baseline; cluster-based permutation test, *P* < 0.05). Similar magnitudes of change were elicited by stimulation of PPN and LDT cholinergic axons (% change in firing rates: PPN, 62.05 ± 7.4; LDT, 52.54 ± 16.2; CINs, −50.17 ± 5.2; one-way ANOVA *F*(2,41) = 28.19, *P* = 0.00001; Bonferroni post hoc analysis: CIN vs. LDT *P* = 0.0001, CIN vs. PPN *P* = 0.0001, LDT vs. PPN *P* = 1.0). Individual data points and mean ± SEM are shown. *P < 0.05. All experiments have been replicated at least three times.

We next tested the effects of stimulating PPN/LDT cholinergic axons on the activity of different types of striatal neurons and compared the effects to the responses elicited by stimulating CINs axons (Figs. 2 and 3). Cholinergic neurons of the striatum, PPN or LDT were transduced with channelrhodopsin-2 (ChR2) in ChAT::Cre+ rats (Fig. 2a, b) in order to produce a differential optogenetic activation of midbrain or CINs axons. The spontaneous activity of individual striatal neurons was first recorded in vivo in anesthetized animals, then cholinergic axons were stimulated with blue light to activate ChR2 through an optic fiber that was integrated within the recording glass pipette electrode to reduce the spread of the light; the recorded neurons were subsequently labeled with neurobiotin using the juxtacellular method (Fig. 2c, f, i; Fig. 3a, d, g;^19^) and their neurochemical nature was confirmed using immunohistochemistry. During urethane-induced slow-wave activity, different types of striatal neurons exhibited different firing rates (basal firing rate, SPNs: 1.19 ± 0.13 Hz, *n* = 91; CINs 2.86 ± 0.37 Hz, *n* = 53; parvalbumin [PV]-expressing interneurons: 7.24 ± 1.33 Hz, *n* = 28), in agreement with previous studies (only neurons recorded during slow-wave activity were included^23^). We confirmed that the expression of ChR2 in CINs increases their firing discharge during the presentation of blue light (Supplementary Fig. 2A–D). The same light stimulation affected neither the firing rate of neurons expressing the reporter alone (Supplementary Fig. 2E–H) nor the activity of their postsynaptic targets (data not shown). Activation of ChR2-expressing cholinergic axons had distinct effects on different subtypes of striatal neurons, and only those recorded/labeled neurons within areas of YFP-transduced axons were observed to respond to the stimulation (Figs. 2d, g, j and 3b, e, h; Supplementary Fig. 2H); for this reason, PPN axon stimulation only produced responses in the anterior dorsolateral striatum and LDT axon stimulation only produced responses in the anterior dorsomedial striatum (Supplementary Fig. 3). Some neurons were recorded in the nucleus accumbens but were not considered in the analysis (Supplementary Fig. 2I–J). In SPNs, all three sets of cholinergic axons produced a significant reduction in the firing rate during the presentation of blue light (Fig. 2e, h, k; cluster-based permutation test, 200 permutations, *P* < 0.05). Furthermore, there was no significant effect on the magnitude of SPN inhibition between PPN, LDT, and CINs. However, the latency of the inhibition was shorter for CINs (0.19 s after laser onset, defined by the inhibition period identified by the permutation test, see blue bar in Fig. 2k) compared to either of the midbrain sources, and LDT effects were shorter in duration when compared to PPN or CINs (4.03 s for PPN after laser onset, gray bar (Fig. 2e), and 1.92 s for LDT after laser onset, red bar (Fig. 2h)). Repeated pulses of blue light stimulation in the PPN produced consistent effects across trials and revealed a long-lasting inhibition spanning several seconds (Supplementary Fig. 4). Thus, optogenetic stimulation of cholinergic axons, regardless of the axon origin (i.e. PPN, LDT, and CINs), produced a significant decrease in the firing rate of SPNs.

In contrast to SPNs, the effects on CINs were different depending on the origin of the cholinergic axons: ChR2 stimulation produced excitation of CINs if the axons originated in the midbrain (Fig. 3a, c, d, f) but inhibition if the axons originated in the striatum (Fig. 3g, i; cluster-based permutation test, 200 permutations, *P* < 0.05, *n* = 10). Notably, the latency for producing a significant excitation by PPN afferents was shorter than that provided by LDT afferents (same analysis as above; 0.57 s for PPN after laser onset, gray bar, Fig. 3c, and 3.07 s for LDT after laser onset, red bar, Fig. 3f). Furthermore, the inhibition of CINs following the stimulation of axons belonging to neighboring CINs was shorter in latency (0.19 s after laser onset, blue bar, Fig. 3i) and similar to their inhibitory effects on SPNs. In contrast to the duration of the inhibition in SPNs, which was observed to extend beyond the end of the light stimulation period, the effects on CINs (both excitation and inhibition) were shorter and largely restricted to the stimulation period.

**Fig. 4:**
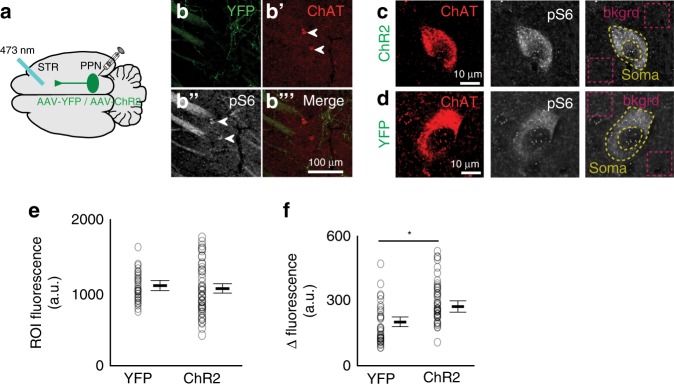
Increase in the phosphorylation of serine 240–244 in CINs following activation of midbrain cholinergic axons. **a** Schematic of the experiment delivering optogenetic stimulation in striatal neurons of ChAT::Cre rats injected with AAV-DIO-ChR2-YFP (five rats) or AAV-DIO-YFP (controls; five rats) in the midbrain. **b** Low magnification confocal images showing the presence of transduced cholinergic axons from the midbrain in the vicinity of CINs **b**′ that are co-expressing pSer240-244 (pS6) **b**″. **c**, **d** High magnification confocal images showing pSer240-244 intensity in CINs following blue laser stimulation of ChR2/YFP-transduced **c** or YFP-transduced (**d**, controls) cholinergic midbrain axons in the striatum. No differences were observed in the expression of pSer240-244 signal in the background (**e**; unpaired two-sided *t*-test, *P* = 0.8033), whereas a significant difference in fluorescence was found between the background and the soma (**f**; 165 neurons, unpaired two-sided *t*-test, *P* = 0.00001; see “Methods”) following laser stimulation of YFP or ChR2-YFP-expressing cholinergic midbrain axons in the striatum. Individual data points and mean ± SEM are shown. *P < 0.05.

To further characterize the excitatory effect of the cholinergic midbrain on CINs, we analyzed changes in the metabolic activity of CINs^24^ following PPN/LDT stimulation (Fig. 4a–c). Blue light stimulation increased the immunohistochemical detection of the phosphorylated ribosomal protein S6 in CINs around the tip of the optic fiber (Fig. 4e, f) but not if the axons only expressed the reporter. Our results suggest that midbrain cholinergic neurons are able to activate CINs by increasing their firing rate and increasing their metabolic activity.

Neurons expressing PV did not show a significant effect to the stimulation of CINs or midbrain cholinergic axons (Supplementary Fig. 5). While a fraction of PV neurons showed an inhibitory response during the laser stimulation, this response was not consistent across recordings and showed large variability.

Altogether, the results from the in vivo electrophysiology demonstrate that midbrain cholinergic neurons have a differential effect on the dynamics of striatal neurons and their firing rates, inhibiting SPNs and exciting CINs. Furthermore, our data suggest that two functionally distinct sources of ACh operate in the striatum, and that the modulation of CINs seems to be at the center of these differences.

### Striatal circuit effects of midbrain cholinergic activation

**Fig. 5:**
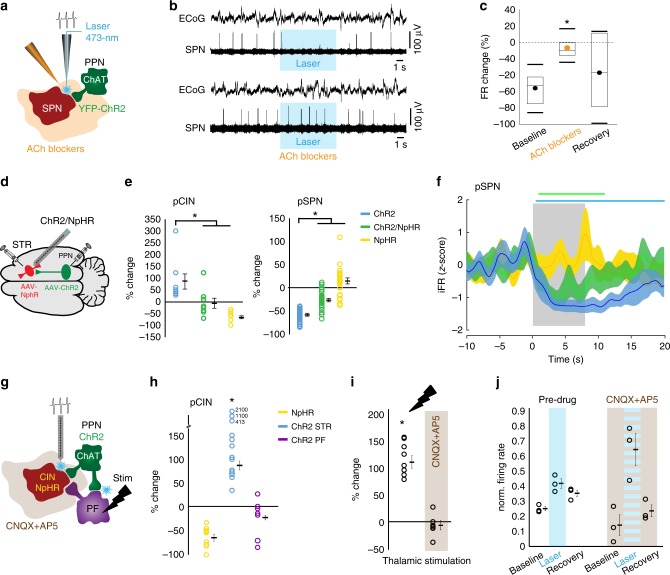
Optogenetic and pharmacological dissection of PPN and LDT effects. **a** Schematic of the in vivo optogenetic and acetylcholine antagonists experiment design. **b, c** pSPNs decreased their activity during ChR2-PPN activation but not in the presence of cholinergic blockers (*n* = 7 neurons, *n* = 7 rats; one-way ANOVA *F*(2,20) = 5.24, *P* = 0.0161; Bonferroni post hoc analyses: before*/*during *P* = 0.014, before*/*after *P* = 0.611, during*/*after *P* = 0.221). **d** In vivo optogenetic experiment design where ChR2 was transduced in the midbrain and halorhodopsin (NpHR; AAV-DIO-NpHR3.0-mCherry) in the striatum (*n* = 7). **e** pCINs (*n* = 8) and pSPNs (*n* = 33) firing rate changes during stimulation of ChR2, NpHR, or both. pCINs activity was significantly higher during ChR2 activation than during NpHR activation or both (ChR2: +83.10 ± 32.09%; NpHR: −71.19 ± 5.61%; ChR2/NpHR: −6.97 ± 22.22%; one-way ANOVA *F*(2,23) = 11.59, *P* = 0.0004). pSPNs activity was significantly lower during ChR2 compared to NpHR and ChR2/NpHR (ChR2: −85.07 ± 1.38%; ChR2/NpHR: −61.40 ± 15.46%; paired *t*-test, *t*(42) = −9.744, *P* = 2.48 × 10^−12^). **f** pSPNs normalized instantaneous firing rate using the same stimulation protocols (color lines represent significant differences; cluster-based permutation test, *P* < 0.05). pSPNs inhibition by midbrain axons seems to partly depend on CINs activity. **f** Circuit mechanisms underlying the excitatory effects of PPN on CINs (*n* = 8 rats). ChR2 was activated in either the striatum or the parafascicular thalamic nucleus (PF), and glutamate antagonists (CNQX + AP5) were locally delivered. **h** Optogenetically inhibited pCINs increased their firing following ChR2-stimulation in the striatum but not the PF, thus ruling out antidromic activation (%-change in firing rate: NpHR, −64.6 ± 7.18, *n* = 13; ChR2-STR, 84.49 ± 9.35, *n* = 11; ChR2-PF, −22.9 ± 4.88, *n* = 7; univariate ANOVA *F*_(2,28)_ = 70.102, *P* < 0.001; Tukey HSD post hoc: ChR2-STR vs. ChR2-PF *P* < 0.001). **i** Neurons activated by PPN axons were unable to respond to electrical pulses in the PF in the presence of glutamate blockers (%-change in firing rate pre-drug: 107.71 ± 12.98, *n* = 8; post-drug: −6.39 ± 8.27, *n* = 6; paired two-tailed *t*-test *t*(5) = 5.198 *P* = 0.003), confirming glutamate blockers efficiency. **j** Activated striatal neurons were still responding to PPN stimulation in the presence of glutamate blockers (*n* = 3), suggesting that the excitatory responses are independent of glutamate transmission (paired two-tailed *t*-test; pre-drug: *t*(2) = −3.828 *P* = 0.031; CNQX + AP5: *t*(2) = −2.904 *P* = 0.051). Individual data points and mean ± SEM are shown. *P < 0.05. All experiments have been replicated at least three times.

To identify the contribution of ACh to the PPN/LDT-mediated inhibition of SPNs, we used a combined approach using in vivo electrophysiology, pharmacology, and optogenetics in urethane-anesthetized rats (*n* = 7), where a small cannula (to deliver ACh receptor antagonists) and an optic fiber (to deliver blue light) were attached to an extracellular tungsten electrode (Fig. 5a). We defined the putative nature of recorded neurons based on their firing rate, action potential duration and coefficient of variation (CV), as previously described^23,25^. Individual putative SPNs (pSPNs; firing rate <1 Hz and action potential <2 ms) were recorded during their baseline activity and subsequently during the stimulation of PPN/LDT terminals. If pSPNs responded to the stimulation, a cocktail of nicotinic and muscarinic antagonists was infused and the response to the laser was tested again 15 min later (Fig. 5b). pSPNs that decreased their firing rate as a result of the stimulation (−56.25 ± 7.47%) showed an abolished response to PPN/LDT stimulation in the presence of cholinergic blockers (−7.2 ± 4.9%; 15 min after drug delivery; Fig. 5c). The inhibition to the laser was partially recovered ~45 min after the drug application (−36.15 ± 16.37%). These results reveal that PPN/LDT cholinergic neurons inhibit the activity of SPNs through an ACh-mediated mechanism.

Given the direct evidence of synaptic connectivity of PPN/LDT axons with CINs (Fig. 1h–k) and the longer latency of PPN/LDT to inhibit SPNs when compared to CINs, we then examined whether the inhibitory effects of PPN/LDT on pSPNs could be mediated by their excitatory effects on CINs. We first transduced PPN/LDT cholinergic neurons with ChR2 and transduced CINs with halorhodopsin (NpHR; Fig. 5d). Then we recorded the activity of striatal neurons (Supplementary Fig. 6) using high-density electrodes and delivered alternating trains of blue and yellow light, or their combination, in order to activate ChR2 and/or NpHR. Only neurons recorded within the vicinity of midbrain (YFP) and CIN axons (mCherry), as determined by the tracks of the electrode (Supplementary Fig. 6A), were used for the analysis (*n* = 132 single units). In line with our results above, putative CINs (pCIN, average firing rate: 1.93 ± 0.38 Hz; Supplementary Fig. 6C) were activated by blue light (ChR2; PPN/LTD axons), strongly inhibited by yellow light (NpHR expressed in CINs) and failed to activate during concurrent blue and yellow light stimulation (Fig. 5e, f, Supplementary Fig. 6D). In addition, pSPNs (average firing rate: 1.09 ± 0.09 Hz; Supplementary Fig. 6B) were inhibited by blue light (ChR2 in PPN/LTD axons; cluster-based permutation test, 200 permutations, *P* < 0.05; Fig. 5e, f). No significant effects on the firing of pSPNs were observed during yellow light stimulation (NpHR in CINs; 11.019 ± 57.61%; cluster-based permutation test, 200 permutations, *P* = 0.765). During concurrent blue/yellow light stimulation, the inhibitory response of pSPNs was significantly reduced (Fig. 5e, f) and the duration was shortened (compare blue and green bars obtained from the cluster-based permutation test, 200 permutations, *P* < 0.05), although it was not entirely abolished. Our results suggest that PPN/LDT cholinergic axons inhibit SPNs through a combined effect that is partly mediated by the excitation of CINs.

To gain further insights into the nature of the ACh effects on CINs, we used an ex vivo approach. ChAT::Cre mice were transduced with ChR2 in the striatum or PPN/LDT, and the activity of CINs was recorded during the delivery of blue-light pulses. Stimulation of CINs produced inhibitory responses in neighboring non-transduced CINs (Supplementary Fig. 7A, B) and this inhibition was abolished in the presence of bicuculline or DHβE (Supplementary Fig. 7B), in line with the evidence of a disynaptic mechanism mediated by GABAergic interneurons. Stimulation of PPN/LDT terminals produced inconclusive results (Supplementary Fig. 7D–G). To circumvent this limitation, we instead used a pharmacological approach, where carbachol was puffed onto striatal CINs. Because optogenetic activation of CINs produces inhibition in neighboring CINs (as shown both in vivo (Fig. 3i) and in vitro (Supplementary Fig. 7B)), any excitatory responses resulting from ACh release must be driven by PPN/LDT terminals. Local administration of carbachol to CINs in the presence of glutamate blockers (CNQX and AP5) and a muscarinic blocker (atropine) produced large excitatory currents in 4/11 CINs recorded (Supplementary Fig. 7C^26^). This suggests that PPN/LDT terminals may potentially recruit the activity of CINs through the activation of nicotinic receptors.

Because nicotinic receptors are located in thalamostriatal terminals, which are a major source of glutamate to the striatum, and because the parafascicular nucleus of the thalamus (PF) receives prominent cholinergic innervation from the midbrain^27–29^, we next investigated whether thalamic afferents mediate the excitatory responses in CINs following the optogenetic activation of PPN/LDT cholinergic axons. ChAT::Cre+ rats were transduced with ChR2 in the PPN/LDT and NpHR in the striatum as before, and a combined optic fiber/stimulation electrode device was implanted in the PF (Fig. 5g). In vivo electrophysiology recordings were performed in the striatum using a custom-made optrode with an attached cannula for drug delivery; only neurons that were followed across the different stages of the experiment were included in the analysis. In the first set of experiments, we tested whether a possible antidromic activation of PPN cholinergic axons in the striatum could activate PPN axon collaterals that innervate the PF and in turn activate CINs. Yellow-light pulses were delivered to activate NpHR and optogenetically tag CINs. Blue-light pulses were delivered in the striatum and the thalamus to activate ChR2 (Fig. 5g). We observed that optogenetically tagged CINs were excited only during striatal, but not thalamic, blue light delivery (Fig. 5h), suggesting that PPN cholinergic axons are unlikely to activate PF neurons that in turn innervate CINs. These results suggest that antidromic activation of PPN cholinergic axons does not account for the excitatory responses in CINs. Next, we investigated whether CINs activation was a consequence of nicotinic-mediated presynaptic glutamate release from thalamic terminals. To this end, striatal neurons were first identified on the basis of their excitatory response to PPN/LDT cholinergic axon stimulation and their firing properties, and subsequently tested during electrical stimulation of the PF before and after the striatal infusion of a cocktail of glutamate blockers (CNQX and AP5), which aimed to test the effectiveness of our pharmacological approach (Fig. 5i). Once we were able to confirm that thalamic transmission was blocked, a few of the same striatal neurons were maintained and thus were tested again during PPN/LDT stimulation, but this time in the presence of glutamate blockers (Fig. 5j). Our results reveal that glutamatergic antagonists are unable to abolish the excitatory responses produced by PPN/LDT stimulation. Given that no other sources of excitation in CINs have been identified other than glutamate^30^ and ACh (Supplementary Fig. 7), our results strongly suggest that PPN/LDT cholinergic axons directly activate CINs.

In summary, the data from these experiments show that PPN/LDT cholinergic axons inhibit the activity of SPNs by means of a cholinergic mechanism that is partly dependent on the activation of CINs, and potentially modulate CINs through a nicotinic mechanism.

### Encoding of behavior by cholinergic systems in the striatum

Cholinergic transmission in the striatum has been associated with updating of action-outcome associations. CINs have been shown to facilitate the integration of new learning into old strategies, whereas cholinergic PPN/LDT neurons seem to be involved in behavioral shifting and updating the behavioral state triggered by changing contingencies^21^, thus having a seemingly convergent function. In order to interrogate the contribution of the midbrain cholinergic system in striatal-dependent behavior, we used a chemogenetic strategy to inhibit the local release of ACh in the striatum (hM4Di transduced in cholinergic neurons of ChAT::Cre+ rats)^31^ during the acquisition of an instrumental lever-press task designed to interrogate different action strategies (i.e., habitual and goal-directed) associated with distinct striatal domains (i.e., dorsolateral and dorsomedial)^32–35^. Chemogenetic-induced reduction in the firing of cholinergic neurons was verified in slice recordings (Supplementary Fig. 8). Validation of the behavioral effects of DREADDs in the PPN was obtained by reproducing the reported effects of lesions in an instrumental task^36^ in a separate cohort of animals (Supplementary Fig. 9). No overall motor effects associated with the chemogenetic inhibition of PPN cholinergic neurons were observed (Supplementary Fig. 10). Thus, ChAT::Cre+ and WT rats were transduced with hM4Di into the PPN, LDT, dorsolateral or dorsomedial striatum (Supplementary Fig. 11A). Bilateral cannulas were implanted in the dorsolateral (for DLS and PPN groups) or dorsomedial striatum (for DMS and LDT groups) for intracerebral delivery of clozapine-N-oxide (CNO; Supplementary Fig. 11D). Before each training session, rats received intrastriatal infusions of CNO (1.5 µM, 250 nl, 30 min before), which was calculated to diffuse 300–500 µm from the tip of the cannula (Supplementary Fig. 11D, E). Rats were trained to press a lever to obtain a reward in a fixed ratio schedule (one press, one reward; FR1) and then trained in a random ratio (RR) schedule to investigate the formation of goal-directed behavior. Following devaluation (see below), rats were retrained in FR1 and then trained in a random interval (RI) schedule to investigate the formation of habitual behavior^32^ (Supplementary Fig. 12A–C).

**Fig. 6:**
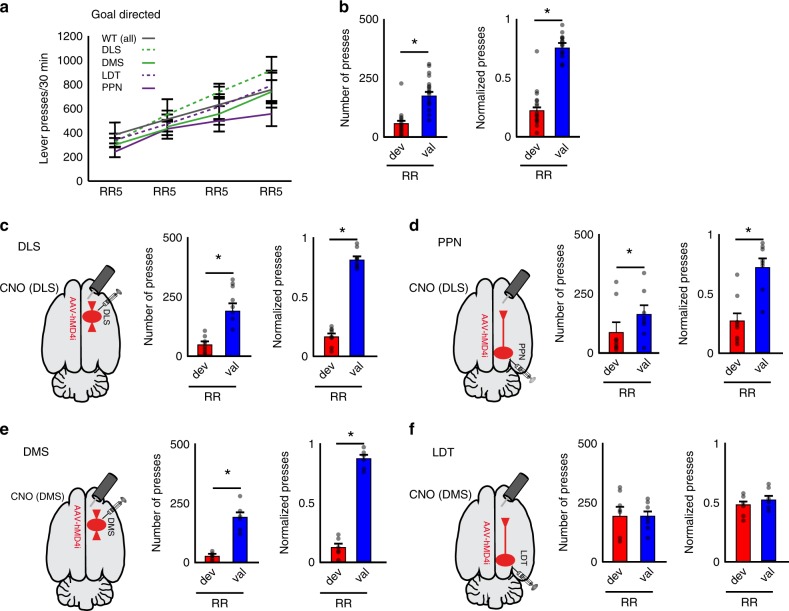
Blocking of LDT cholinergic transmission in the striatum impairs goal-directed action selection. **a** Lever pressing during acquisition of goal-directed behavior shows no significant difference in the interaction between group and day of training (two-way ANOVA group [WT (all), DLS, DMS, LDT, PPN]×day: *F*_group_(4,180) = 2.60, *P* = 0.0375; *F*_day_(3,180) = 22.48, *P* = 0.00001; *F*_interaction_(12,180) = 0.46, *P* = 0.9343; *n* = 46 rats). **b** Number of presses and normalized lever presses during outcome devaluation testing across valued (val, blue) or devalued (dev, red) states in random ratio schedule (RR; goal-directed) in control animals. The number of presses was significantly different across sessions in all control groups (mixed ANOVA group × session[valued, devalued]; *F*_group_(3,16) = 0.65, *P* = 0.59; F_session_(1,16) = 37.035, *P* = 0.000016; *F*_interaction_ (3,16) = 0.83766, *P* = 0.492824) as well as the normalized lever presses (mixed ANOVA group × session: *F*_session_(1,16) = 71.70, *P* = 0.00001, *F*_interaction_(3,16) = 1.43, *P* = 0.2706). Control groups were then pooled into one single group. **c**–**f** ChAT::Cre animals were injected in the DLS **c**, PPN **d**, DMS **e** or LDT **f**. During RR, significant differences in goal-directed devaluation were observed in the number of presses (mixed ANOVA group × session: *F*_group_(4,45) = 2.315, *P* = 0.07, *F*_session_(1,45) = 76.13, *P* = 0.00001; *F*_interaction_(4,45) = 6.31, *P* = 0.0004) and the normalized lever presses (mixed ANOVA group × session[valued, devalued], *F*_session_(1,45) = 138.39, *P* = 0.00001, and *F*_interaction_(4,45) = 7.44, *P* = 0.0001). Post hoc comparisons show differences in all groups except LDT (see text for values), suggesting that inhibition of LDT axons prevents animals from developing goal-directed behavior. Individual data points and mean ± SEM are shown. *P < 0.05. All experiments have been replicated at least three times.

**Fig. 7:**
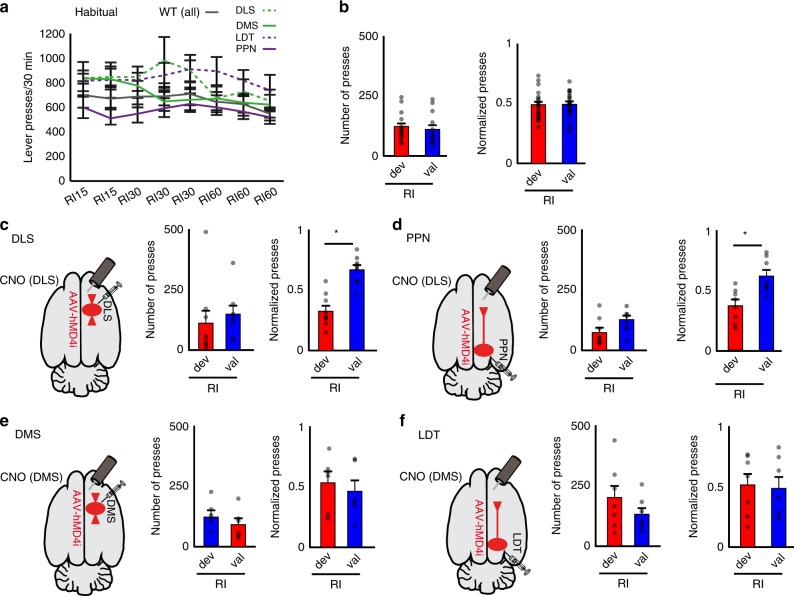
Blocking of cholinergic transmission in the dorsal striatum impairs habitual action control. **a** Lever pressing during acquisition of habitual behavior shows no significant differences between groups (two-way ANOVA group × day: *F*_group_(4,359) = 2.30, *P* = 0.0581, *F*_day_(7,359)=0.98, *P* = 0.4437; *F*_interaction_(28,359)=0.36, *P* = 0.9991; *n* = 46). **b** Number of presses and normalized lever presses during outcome devaluation testing across valued (val, blue) or devalued (dev, red) states in random interval schedule (RI; habitual) in control animals. Neither the number of presses (mixed ANOVA: *F*_group_ (3,16) = 1.16, *P* = 0.355; *F*_session_(1,16) = 0.16, *P* = 0.6957; *F*_interaction_(3,16) = 0.80, *P* = 0.5124) nor the normalized lever presses (mixed ANOVA group × session: *F*_session_(1,16) = 0.001, *P* = 0.9846, *F*_interaction_(3,16) = 0.1, *P* = 0.9576) showed significant differences. Control groups were then pooled into one single group. **c**–**f** ChAT::Cre animals were injected in the DLS **c**, PPN **d**, DMS **e** or LDT **f**. During RI, there were no differences in the number of presses (mixed ANOVA: groups × session [valued vs. devalued], *F*_group_(4,45) = 0.951, *P* = 0.444, *F*_session_(1,45) = 0.73, *P* = 0.3975, *F*_interaction_(4,45) = 2.31, *P* = 0.0719) but there was a significant interaction in the normalized number of presses (mixed ANOVA groups × session: *F*_session_(1,45) = 4.10, *P* = 0.0488; *F*_interaction_(4,45) = 3.0, *P* = 0.0282). Post hoc comparisons show a significant devaluation in DLS and PPN groups (see text for values), but not in the rest of the groups, suggesting that inhibition of PPN and DLS CINs axons prevents animals from developing habitual learning. Individual data points and mean ± SEM are shown. *P < 0.05. All experiments have been replicated at least three times.

The control group consisted of WT rats receiving the same manipulations (i.e., viral injection, cannulation, and CNO delivery) and training as the experimental group. Animals showing histological signs of striatal lesions in any group were not considered for further analysis. No differences due to CNO (versus saline) infusion were observed in any group [WT and ChAT::Cre+ rats, each virally transduced in the DLS, DMS, PPN or LDT] in locomotor activity, evaluated as total distance traveled and distance in center of the open field (Supplementary Fig. 12D, E). No changes were detected in sugar consumption either (two-way ANOVA groups × drugs: *F*_group_(4,99) = 1.94, *P* = 0.11, *F*_drugs_(1,99) = 0.001, *P* = 0.96, *F*_interaction_(4,99) = 0.08, *P* = 0.99), suggesting that midbrain cholinergic terminals targeting other structures were not affected^37^. During training, the number of lever presses during RR increased gradually over successive sessions in all groups (Fig. 6a), whereas during RI they remained constant (Fig. 7a)^38,39^. All animals were then tested in an outcome devaluation task, consisting of two counterbalanced sessions carried out over two consecutive days: a ‘valued’ session where rats were fed rat chow but no sugar pellets (the instrumental outcome) immediately before testing (maintaining a high motivational state for the outcome) and a ‘devalued’ session where rats were fed sugar pellets before testing (thus devaluing the instrumental outcome). While goal-directed behavior is expected to be sensitive to the motivational changes of the devalued session, resulting in a reduction in the number of lever presses, habitual behavior is not expected to be affected by pre-exposure to the instrumental outcome and therefore lever pressing in the devalued session should not be significantly reduced^32,33,40^. Thus, control animals (WT) showed a higher number of lever presses in the valued session compared to the devalued session following RR training (Fig. 6b) but no significant differences in the number of lever presses in the valued and devalued sessions following RI training (Fig. 7b). We also calculated the difference between valued and devalued responses as an index that reveals the ability of animals to adjust their responses after training in each schedule (see “Methods”; Supplementary Fig. 13) and observed a strong preference to seek the instrumental outcome in the valued condition compared to the devalued condition following RR training sessions, contrasting with a lack of preference following RI training sessions, thus denoting bias towards either goal-directed or habitual behavior, respectively.

Next, we interrogated the contribution of ACh to this behavior in two striatal regions (dorsolateral and dorsomedial) in the ChAT::Cre animals compared to the WT animals. Because there was no difference in the number of lever presses between control groups (i.e. associated with the brain region targeted, Supplementary Fig. 12B, C), the WT data were pooled into one single control group for the following analyses. We inhibited cholinergic transmission from axons arising in dorsolateral CINs (Fig. 6c), PPN (Fig. 6d), dorsomedial CINs (Fig. 6e), or LDT (Fig. 6f) during training. No effect of group vs. day interaction was observed in the response rates during RR training. After RR training, most animals showed a significant reduction in lever pressing in the devalued session relative to the valued session, similar to the reduction in WT. Post hoc pairwise comparisons (paired two-tailed *t*-test) revealed a significant difference in lever presses (devalued vs. valued) in WT (*P* < 0.0001; Fig. 6b), DLS (*P* < 0.0001; Fig. 6c), DMS (*P* = 0.001; Fig. 6e), and PPN (*P* = 0.0076; Fig. 6d) but not in LDT (*P* = 0.9597; Fig. 6f) groups. The normalized number of presses (see “Methods”) also revealed a significant interaction using a post hoc pairwise test (paired two-tailed *t*-test), which showed significant effects in the WT (*P* < 0.0001), DLS (*P* < 0.00001), DMS (*P* < 0.0001), and PPN (*P* < 0.015) but not LDT (*P* = 0.5728) groups (Fig. 6b–f). Thus, animals in the LDT group showed virtually the same proportion of lever presses during both valued and devalued sessions suggesting reduced expression of goal-directed behavior (Fig. 6f). In other words, when ACh release in the DMS arising from LDT terminals was disrupted during RR training, rats failed to associate the outcome with the instrumental action that produced it and were therefore insensitive to reward devaluation. This effect was evident by the absence of change in the devaluation index in LDT despite the different training conditions (Supplementary Fig. 13).

Finally, following retraining in FR1 in the absence of CNO administration to reach the same criterion as continuous reinforcement (paired two-tailed *t*-test *t*(45) = 0.53, *P* = 0.5965), we tested the effects of cholinergic transmission on habitual training in the same group of animals (Fig. 7). During RI training, no effect of group, day, or interaction was observed in the response rates (Fig. 7a). Animals in the dorsomedial striatum group showed no significant differences in the number of lever presses during the valued and devalued sessions, as controls did, suggesting that habitual behavior encoding remained intact (Fig. 7e); animals in the LDT group showed a similar number of presses than the dorsomedial striatum group but because animals did not develop goal-directed behavior in the previous experiment, it is uncertain whether this had an impact on their performance during habitual behavior, and as a result, these data should be interpreted with caution (Fig. 7f). However, there was a significant interaction in the normalized number of presses, and post hoc pairwise comparisons (paired two-tailed *t*-test) revealed a significant difference of normalized lever presses (devalued vs*.* valued) in DLS (*P* = 0.005; Fig. 7c) and PPN (*P* = 0.035; Fig. 7d), but not for WT (*P* = 0.9833; Fig. 7b), DMS (*P* = 0.7188; Fig. 7e) and LDT (*P* = 0.8832; Fig. 7f). This suggests that rats in the DLS and PPN groups formed an association between lever pressing and reward delivery typical of goal-directed learning that led them to reduce their actions during the devalued session, in contrast to the other groups which showed no effect of devaluation on lever pressing. Thus, reduced cholinergic transmission in the DLS, regardless of its origin (i.e., CINs and PPN), impairs the ability of rats to form habitual behavior (as also observed following DLS lesions^41^), thus revealing that cholinergic neurons from the midbrain have a critical role in normal striatal operations.

## Discussion

Cholinergic transmission in the striatum powerfully modulates striatal output^42^, the activity of striatal interneurons^43–45^, the release of glutamate from cortical and thalamic terminals^46^ and the release of dopamine from mesostriatal terminals^47,48^. We present here detailed evidence of the functionality of a hitherto uncharacterized source of ACh in the striatum originating in the midbrain. We show that cholinergic neurons of the PPN and LDT provide direct innervation of CINs and direct and indirect pathway neurons. We show that PPN and LDT axon terminals inhibit the activity of SPNs while activating CINs, suggesting a circuit mechanism in which PPN/LDT may potentially modulate striatal activity through CINs. Finally, we show that inhibition of cholinergic transmission from either PPN, LDT, or CINs impairs encoding of action strategies, suggesting that cholinergic transmission from the midbrain is necessary for normal encoding of behavior.

Our results show that PPN and LDT cholinergic neurons inhibit the firing of SPNs with a similar magnitude as CINs, suggesting overlapping actions. Because PPN and LDT preferentially contact CINs over SPNs (as supported by both electron microscopy and monosynaptic rabies labeling), and because their latency for activating CINs is shorter than for inhibiting SPNs, we hypothesized that PPN/LDT neurons may be exerting their effects in the striatum partly through their connections with CINs. Despite considerable efforts in attempting to characterize this connection in the slice, we were unable to obtain any postsynaptic response to the optogenetic stimulation of midbrain cholinergic axons in any of the regions tested (Supplementary Fig. 7), including the striatum, the thalamus, locally in the PPN and the ventral tegmental area (VTA^19^), in line with previous reports by ourselves^49^ and others^20^; these technical difficulties are only observed ex vivo and particularly associated with the cholinergic phenotype (see ref. ^50^), likely uncovering a particular susceptibility in PPN cholinergic axons or other unaccounted factors. For this reason, we chose to dissect this circuit in vivo. We then observed that the inhibitory effects of the PPN/LDT over SPNs were significantly reduced but not abolished following the inhibition of CINs and completely abolished following the infusion of ACh blockers. These results suggest that CINs play a key role in the modulation of the striatal output by PPN/LDT, but additional mechanisms are likely to contribute to the cholinergic modulation arising in the midbrain. Such mechanisms may rely on a monosynaptic modulation of SPNs by PPN/LDT cholinergic axons (as shown by our anatomical data in Fig. 1 and the evidence of synaptic contacts with dendritic spines in ref. ^8^) or through other GABAergic interneurons^51^.

The seemingly overlapping effects of cholinergic signaling arising from two different sources raise the question of whether they are conveying different messages or acting in coordination. Several differences between striatal CINs and midbrain cholinergic neurons have been reported in the literature. CINs receive innervation predominantly from cortical areas, including cingulate, secondary motor and primary somatosensory cortices^52^, and thalamic nuclei including the parafascicular and centrolateral^53,54^. Inputs to cholinergic midbrain neurons have not been fully identified, but largely differ from CINs as they predominantly arise in basal ganglia structures, including the SNR and internal GPE^55–57^. In terms of the physiological properties, CINs possess a high-input resistance (200 MΩ)^58^ and have been associated with a spontaneous, tonically active firing mode (3–10 Hz^59^); that is mediated by inward rectifying potassium currents and a depolarization sag that induces rebound spike firing^60^. In contrast, PPN cholinergic neurons show a low firing rate in vitro (2–3 Hz), a very high input resistance (600 MΩ), display an A-current^61^, their firing seems to be modulated by M-currents^62^ and show fast-adaptive firing^15^. In vivo, identified PPN cholinergic neurons have been shown to fire phasically^15,16^. This evidence thus indicates that midbrain and striatal cholinergic cell groups differ in their afferent connectivity and physiological properties, suggesting they are modulated differently by their afferents and that their dynamics are distinct.

Another significant difference stems from electrophysiological recordings of putative striatal and midbrain cholinergic neurons in awake, behaving animals. Tonically active neurons in the striatum encode a pause in their firing rate that is associated with behaviorally relevant salient events^63,64^, which is correlated with the phasic activation of dopamine neurons in mesostriatal systems. Importantly, this pause is often preceded by a phasic increase in firing before the inhibition, mediated in part by thalamostriatal activation^65^ and followed by a rebound excitation. Neurons in the PPN, in contrast, increase their firing rate phasically during sensory cues that predict reward presentation^66^, presumably driving dopamine transients in the striatum. The multiphasic response of CINs during behaviorally relevant salient events suggests the convergence of multiple synaptic drives that shape the burst-pause-rebound dynamics of CINs. The direct connectivity and excitatory nature of the midbrain input onto CINs suggest that PPN/LDT cholinergic neurons contribute to sculpting the response of CINs during behavior. Further experiments are needed to determine the extent of this modulation.

Our data here also suggest that there are intersecting roles of CINs and PPN/LDT neurons in cholinergic-mediated striatal behavior. In the anterior dorsolateral striatum, we revealed that inhibition of cholinergic signaling arising from either CINs or PPN neurons is able to block the encoding of habitual behavior, whereas in the anterior dorsomedial striatum inhibition of cholinergic signaling from LDT neurons is able to block the encoding of goal-directed behavior. Together with our anatomical data showing preferential innervation of PPN and LDT neurons over CINs, these behavioral effects suggest that midbrain cholinergic neurons modulate the activity of CINs during behavioral shaping and action strategy. Similar changes in the outcome of these tasks have been obtained following the interruption of the thalamostriatal projections that target CINs^34^ and corticostriatal projections^33^, or following excitotoxic striatal lesions^41^. All the above suggest that optimal encoding of behavioral information in the striatum is mediated by a series of factors that converge at the level of the CINs; furthermore, it reveals the role of the PPN as a key modulator of striatal activity through CINs.

In line with our findings, the role of the PPN in adaptive behavior and action control has been previously addressed by a series of experiments using lesions or pharmacological manipulations. For example, non-specific PPN lesions impair adaptation to incremental walking speeds in a motor task^67^, affect the assimilation of new strategies with a consequent increase in perseverant responses^36,68^, and decrease the sensitivity to reward omissions^69^, thus denoting a failure in adjusting the behavioral state. Furthermore, pharmacological inhibition of the PPN produces a decrease in the responsiveness to degradation in contingencies between action and outcome, but did not change it if contingencies remain unchanged^70^, in line with findings showing impaired ability of rats to adapt to new strategies when the contingencies changed following inhibition of cholinergic transmission in the striatum^4,71^ or CINs lesions^72^. This body of evidence suggests that interrupting PPN activity has similar effects to those observed following disruption of cholinergic transmission in the striatum and raises the possibility that PPN is mediating such response. In contrast to the PPN literature, less is known about the LDT function. Manipulations of LDT have shown to drive the activity of VTA neurons and modulate positive reinforcement^20,73^. Our results revealed that chemogenetic inhibition of LDT terminals in the DMS blocked encoding of goal-directed behavior, an effect that was not observed by preventing ACh release from CINs in the DMS. These contrasting effects between LDT and CINs suggest that goal-directed behavior is not directly mediated by CINs and that LDT actions may be mediated by other striatal interneurons. In support of this, elimination of CINs in the DMS was shown to enhance behavioral flexibility^74^, while the destruction of thalamic afferents to DMS produced a deficit in goal-directed learning^34^. These studies suggest that thalamic afferents may modulate certain behavioral functions in the DMS through the connectivity with other striatal interneurons^75^, such as the low-threshold spiking interneurons, which were recently found to modulate the acquisition of novel contingencies in a goal-directed task^76^. Such a central role of LDT afferents in the modulation of goal-directed behavior is in agreement with recent evidence supporting its role in reinforcement learning through the modulation of dopamine neurons^19,20,77^. Our experiments here functionally link the intrinsic and extrinsic striatal cholinergic systems by showing that interfering with cholinergic transmission in the striatum, regardless of its origin, has similar functional consequences for action selection.

PPN and LDT have divergent ascending cholinergic projections that converge in the striatum following three different pathways. First, axon collaterals of cholinergic neurons innervate dopamine neurons in the substantia nigra and VTA^78–80^. Activation of this pathway leads to increased firing and bursting activity of dopamine neurons that project to the striatal complex^19^. Second, cholinergic neurons innervate thalamic nuclei that in turn project to the striatum^29,65,81^. Third, our results here reveal that PPN and LDT cholinergic neurons directly innervate both SPNs and CINs, with preferential innervation of the latter. The convergence of three different afferent systems arising from a single cell group in the midbrain puts the PPN and LDT in key positions as modulators of striatal activity and suggests that striatum (whether directly or indirectly) is the main target of cholinergic PPN/LDT projections, as no other PPN/LDT target receives such level of converging afferents from PPN/LDT cholinergic neurons. Furthermore, at least a proportion of these projections originate from the same neurons^8^, potentially indicating the simultaneous activation of dopamine, thalamic, and striatal targets, and suggesting that these converging effects at the striatum level are inextricably linked. In addition, we recently reported the existence of a direct PPN glutamatergic projection to the striatum that exclusively targets interneurons, and in particular CINs, and that elicits feed-forward inhibition in SPNs^50,82^. Altogether, these findings and our present results highlight the key position of CINs for the ascending PPN projections and the concerted net inhibitory effects of PPN neurons over the striatal output.

What is the PPN signaling in the striatum and why is it relevant for behavior? PPN neurons have been shown to have a phasic activation during particular behavioral contexts, such as during Pavlovian conditioning^83^, reward prediction^66,84^ and reward omission^85^. Thus, when these signals are absent because PPN neurons fail to signal a mismatch between expected and real contingencies, the behavior is not updated, creating perseverant responses and failure to integrate new learning with the old learning^21^. The activation of CINs may thus underlie the mechanism by which PPN is able to shape striatal output and block ongoing motor programs at the level of SPNs in order to update the behavioral state and reinforce novel actions.

## Methods

### Experimental subjects

Male and female adult (250–450 g, ~8 weeks old) Long Evans (LE) wild type and ChAT::cre+ rats^86^ were used for all anatomical (20 female/2 male), in vivo electrophysiological (27 female/39 male) and behavioral experiments (18 female/70 male). For electrophysiology and anatomy experiments, animals were maintained on a 12:12 light cycle (light on 07:00) and had ad libitum access to water and food. Animals were group-housed except for recovery following surgery and during behavioral experiments in order to avoid infection and post-implantation complications. Following surgery, all animals received i.p. injections of analgesic (ketofen) and antibiotic (Baytril); no experiments were made 7 days following last drugs administration. For behavioral experiments, animals were maintained on a 12:12 inverted light cycle (light on 19:00) and had ad libitum access to water and food except during behavioral training. All littermates of the same age were allocated to each group based on their genotype and sex. All procedures were performed in accordance with the Society for Neuroscience policy on the use of animals in neuroscience, with the approval of the Rutgers University Institutional Animal Care and Use Committee and in accordance with the NIH Guide to the Care and Use of Laboratory Animals, and the Animals (Scientific Procedures) Act, 1896 (UK), under the authority of Project License approved by the Home Office. For all experiments, all efforts were made to minimize the number of rats used and any possible discomfort.

### Stereotaxic surgery

All stereotaxic injections were performed during deep isoflurane anesthesia (2–4% in O_2_; Isoflo, Schering-Plough, Garden City, UK). For all experiments, animals were placed in a stereotaxic frame (Kopf Instruments), the skull surface was exposed and rats were injected with a virus (Table 1) at specific coordinates (Table 2) using a nano-syringe (1 µl, Hamilton Instrument, product 7001). Injections were made at a rate of 30–40 nl/min and remained in place for 10 min after the end of the infusion. For single-unit juxtacellular recordings, four groups of male and female ChAT::cre+ rats were used and received an injection of AAV2-EF1a-DIO-ChR2-YFP (PPN: *n* = 11; LDT: *n* = 13; CIN: *n* = 16) or AAV2-EF1a-DIO-YFP (controls, PPN: *n* = 6, CIN: *n* = 6). For high-density electrophysiology experiments, male and female ChAT::cre+ rats (*n* = 6) received an injection of AAV2-EF1a-DIO-ChR2-YFP in both the PPN and the LDT (two separate injections) and AAV5-EF1a-DIO-NpHr3.0-mCherry in the rostral region of the dorsal striatum. For anatomical mapping, female ChAT::cre+ (*n* = 4) were injected in the brainstem with AAV2-EF1a-DIO-eYFP and in the striatum with AAV5-EF1a-DIO-mCherry. For in vivo electrophysiology/pharmacology experiments, male ChAT::cre+ rats (*n* = 7) received an injection of AAV2-EF1a-DIO-ChR2-YFP in the brainstem (two separate injections in PPN and LDT). For electron microscopy experiments, female ChAT::cre+ rats (*n* = 4) were injected in the brainstem with AAV2-EF1a-DIO-YFP. For experiments assessing the expression of a transcription factor, female Chat::cre+ were injected in the brainstem with AAV2-EF1a-DIO-ChR2-eYFP (*n* = 6) or AAV2-EF1a-DIO-YFP (*n* = 4). For behavioral experiments, ChAT::cre+ and WT rats were bilaterally injected with AAV2-hSyn-HA-hM4D(Gi)-DIO-mCherry in the PPN (ChAT::cre+ *n* = 9; WT *n* = 5), in the LDT (ChAT::cre+ *n* = 7; WT *n* = 5), in the dorsomedial striatum (DMS; ChAT::cre+ *n* = 6; WT *n* = 5) and in the dorsolateral striatum (DLS; ChAT::cre+ *n* = 8; WT *n* = 5), and bilaterally implanted in the DMS or DLS with a custom-made 26-gauge stainless steel cannula. For retrograde transsynaptic labeling targeting CINs, ChAT::cre+ rats were used (*n* = 4) and were injected with AAV-DIO-mCherry-TVA and AAV-DIO-RG (ΔG) (helper virus) in the DLS and DMS. Two weeks after injections, animals were injected with pseudotyped rabies virus RVdG-YFP in the same coordinates as the helper virus. For retrograde transsynaptic labeling targeting direct-pathway and indirect-pathway SPNs, animals received an injection of CAV2-Cre in the substantia nigra pars reticulata (SNr; *n* = 3) or the external GPE (*n* = 3), respectively, followed by injection of AAV-DIO-mCherry-TVA and AAV-DIO-RG (ΔG) (helper virus) in the DLS or DMS. A different group of animals were injected with the AAV2-EF1a-IRES-WGA-Cre^87^ in order to express the wheat germ agglutinin (WGA)-Cre fusion protein in the SNR or GPE, which will be retrogradely transported to the striatum, or directly into the striatum (to label all striatal neurons, including interneurons). This was followed by helper virus injections into the striatum as above. Two weeks after injections, the pseudotyped rabies virus RVdG-YFP was injected at the same coordinates as the helper viruses. All animals used for rabies virus tracing were kept in a BSL2 facility and were perfused 7 days following the last surgery.

**Table 1:** Viral constructs.

Name	Construct	Titer (vg/ml)	Source
eYFP	AAV2-DIO-EF1a-eYFP	1 × 10^13^	UNC vector core
eYFP-ChR2	AAV2-DIO-EF1a-eYFP-ChR2	1 × 10^13^	UNC vector core
mCherry-NpHR3.0	AAV5-DIO-EF1a-mCherry-NpHr3.0	1 × 10^13^	UNC vector core
mcherry	AAV5-DIO-EF1a-mCherry	1 × 10^13^	UNC vector core
TVA	AAV5-Flex-TVA950-EF1a-mCherry	1 × 10^12^	UNC vector core
ΔG	AAV8-Flex-RG-CAG	1 × 10^12^	UNC vector core
RVdG	SADΔG-GFP(EnvA)	1 × 10^8^	Salk vector core
CAV2-cre	CAV2-cre	6 × 10^12^	IGMM vector core
WGA-cre	AAV-EF1A-IRES-WGA-Cre	6 × 10^12^	UNC vector core
DREADD	AAV2-hSyn-hM4D(Gi)-DIO-mCherry	3 × 10^12^	UNC vector core

**Table 2:** Coordinates and volumes list.

Structure	Coordinates (mm)			Volume (nl)	Rate (nl/min)
	AP	ML	DV		
PPN	−7.30	1.80	−7.50	400	40
LDT	−8.50	0.90	−6.00	300	30
DMS	0.50	1.80	−4.5/−3.5	450	40
DLS	0.50	3.00	−4.5/−3.5	450	40
DS	0.50	2.60	−4.5/−3.5	450	40
SNr	−5.80	1.80	−7.50	500	40
GPE	−1.30	3.00	−6.5	300	30

### Juxtacellular recordings

Anesthesia was induced with 4% v/v isoflurane (Schering-Plough) in O_2_, and maintained by injection of urethane (1.3 g/kg, i.p.; ethyl carbamate, Sigma, Poole, UK). Urethane is a stable anesthetic that preserves neuronal activity which at anesthetic concentrations has modest effects on neurotransmitter-gated ion channels^88^. Supplemental doses of ketamine (35 mg/kg, i.p.; Ketaset, Willows Francis, Crawley, UK) and xylazine (6 mg/kg i.p.; Rompun, Bayer, Germany) were given as required throughout the experiment. Body temperature was maintained at 38 °C using a thermistor controlled heating pad. After local skin anesthesia by subcutaneous injection of Marcaine (0.25%), the animals were placed in a stereotaxic frame (Kopf). A subcutaneous incision was made to expose the skull. Then, craniotomies were made for the electrocorticogram (ECoG, bilaterally from bregma: AP + 3.0 mm, ML + 2.5 corresponding to the somatosensory cortex) and a reference (above the right cerebellum). A craniotomy was made above the striatum and the dura mater was gently removed to allow the passage of the recording pipette. The exposed brain surface was kept moist with sterile saline (0.9% NaCl) throughout the experiment.

The ECoG was recorded using 1 mm diameter stainless steel screws and referenced to a steel screw above the cerebellum. The ECoG signals were band-pass filtered at 0.3–1000 Hz (−3 dB limits), amplified 2000 times (DPA-2FS filter/amplifier; Scientifica, Harpenden, UK) and digitized online at 2.5 kHz. The ECoG was used to monitor the depth of anesthesia. Extracellular recording of action potentials of individual striatum neurons was made using custom-made glass opto-micropipettes (1.5 mm O.D.; 1.17 mm I.D Harvard Apparatus; 15–25 MΩ impedance measured in the cortex, tip diameter ~1.5 µm, with a 105 µm optic fiber, 0.22NA multimode, Thorlabs) filled with 1.5% w/v Neurobiotin (Vector Laboratories Ltd., Peterborough, UK) in 0.5 M NaCl. The optic fiber was inserted and fixed in the glass pipette and the other side was connected to a class IIIb blue laser (473 nm; LaserGlow Tech., Canada) in order to provide a minimum power of 5 mW at the tip of the glass pipette. The output power was tested between penetrations and readjusted if needed. Signals from the glass micropipettes were band-pass filtered at 0.3–5000 Hz (NL125: Digitimer), amplified 10-fold through the active bridge circuitry of an Axoprobe-1-A amplifier (Molecular Devices Corps., Sunnyvale, CA), AC-coupled and amplified a further 100-fold (NL-106 AC-DC Amp: Digitimer Ltd., Welwyn Garden City, UK), and digitized online at 17.5 kHz. Data were acquired and stored using an analog-to-digital converter (Power 1401: Cambridge Electronic Design, Cambridge, UK) connected to a PC running spike2 (ver 7; Cambridge Electronic Design). The whole of the striatum was scanned with the opto-micropipette for spontaneously firing neurons. When action potentials were detected, a minimum of 5 min of basal firing were recorded to establish a mean baseline firing rate and spontaneous discharge pattern. The activity of striatal neurons was recorded during optical stimulation of PPN, LDT, or CINs afferents using a train of light stimulation (8 s, 80 pulses, 10 Hz, 50 ms ON/OFF). Train pulses were repeated at least twice, with a minimum interval of 1 min between each. At the end of the recording, a microiontophoretic current was applied to the neuron to label it with Neurobiotin^89^. To ensure discrimination between neurons during the histological analysis (see below), a maximum of eight neurons were recorded per animal with a distance of at least 400 µm in all axes (*X*, *Y*, *Z*). Following at least 2 h of diffusion time, the animals were given a lethal dose of ketamine and intracardially perfused. Brains were stored in PBS at 4 °C until sectioning.

### High-density extracellular recordings and optogenetics

ChAT::cre+ rats injected with AAV-DIO-ChR2-YFP in the brainstem and AAV-DIO-NpHR-mCherry in the striatum were anesthetized, as described above. Anesthesia was maintained by a urethane injection (1.3 g/kg, i.p.). Body temperature was maintained at 38 °C. Animals were placed in a stereotaxic frame and the skull exposed. A small craniotomy was made above the striatum and the dura was gently removed to allow passage of the recording electrodes. Extracellular activity was recorded in the striatum using a 16-channels silicon probe with an optical fiber mounted 200 µm above the upper-most recording site (Neuronexus, OA16-10mm-100-177). Signals were amplified, digitized continuously at 20 kHz using a head-stage directly attached to the probe (RHD2000, Intan Technology). The fiber attached to the probe was coupled to an optic collimator (CFC-11×-c, Thorlabs) focusing a 473-nm laser (15 mW, OEM) and a 635-nm laser (6 mW, OEM). Laser power was adjusted to minimize optical artifacts. Once one or more units were identified, a 2-to-5 min baseline recording was recorded and then neurons received blue stimulation (10 Hz, 50 ms, 8 s duration), yellow stimulation (continuous 8 s duration) or both synchronized. All stimulations were repeated at least three times, only consistent responses were considered further. Recording files were converted into Spike2 format (CED), band-pass filtered between 300 and 5000 Hz and sorted into single units using principal component analysis. Light responses were determined as the variation of the firing rate during laser stimulation (Hz), percentage of variation compared to the 20 s prior to stimulation (baseline) or the standard deviation from the mean firing rate during baseline activity (*z*-score) and compared between stimulation parameters (blue, yellow or both). Following recordings, animals were perfused and their brain processed. In order to locate the recording site, the back of the silicon probes was stained with Dil (Invitrogen, D3911). Only recordings within a region where both ChR2-YFP and NpHR-mCherry fluorescent signals were overlapping were used for analyses. pSPNs were defined by their physiological properties (see below) and their inhibitory response to blue light (as observed during juxtacellular recording); putative CINs were defined by their physiological properties (see below) and their inhibitory response to yellow light (NpHR expression; decrease of at least 80% of their firing rate over a minimum of two laser stimulations).

### In vivo pharmacology

An in vivo pressure drug-delivery method combined with optogenetic stimulation was adapted from previous reports^19^ to allow simultaneous recording, laser stimulation and drug delivery to pSPNs in anesthetized rats. Custom-made tungsten electrodes (1 MΩ, AM-System #573220) were combined with an optic fiber (200 µm, 0.50NA, Thorlabs) and a stainless-steel tube for drug delivery (87 µm O.D., Small Parts Inc.) that was located ~200–400 µm from the recording site. The optic fiber was connected to a blue laser (3 mW, LaserGlow Tech.). The cannula was connected to a precision syringe (0.5 µl, Hamilton Company) for delivery of 100 nl of nicotinic and muscarinic antagonists (methyllycaconitine (MLA) 20 mM, dihydro-β-erythroidine (DHβE) 40 mM, atropine 40 mM, and mecamylamine 100 μM), as previously described^19^. The recording electrode was connected to an Intan RHD2000 amplifier (20 kS/s) and the signal was referenced to an anchor screw located above the cerebellum. Signals from the electrodes were band-pass filtered at 500–5000 Hz and analyzed using Spike2 software (Cambridge Electronic Design (CED)). Only one recording per animal was performed in order to avoid the effects of the drugs not washing out. Only neurons that responded to the laser stimulation and that were conserved throughout the entire recording were analyzed.

For the experiments dissecting the contribution of the thalamus to the optogenetic stimulation of midbrain cholinergic axons, ChAT::cre+ rats were injected with AAV-DIO-ChR2-YFP in the midbrain and AAV-DIO-NpHR-mCherry in the striatum, and were additionally implanted with custom-built concentric electrodes and an optic fiber into the parafascicular nucleus of the thalamus (PF). Extracellular activity was recorded in the striatum as described above using a 16-channels silicon probe with an optical fiber mounted 200µm above the upper-most recording site (Neuronexus, OA16-10mm-100-177). Further, a 24-gauge FEP polymer catheter was mounted to the silicon probe. Before recording, this tube was back-filled with 6-cyano-7-nitroquinoxaline-2,3-dione (CNQX, 1 mM) and 2-amino-5-phosphonopentanoic acid (AP-5, 50 mM) and connected to a precision syringe (10 µl, Hamilton Company), in turn, mounted into a microsyringe pump. Upon identifying units, neurons received yellow light stimulation (continuous 5 s duration) and then blue light stimulation (10 Hz, 50 ms, 5 s duration). Blue light was also delivered through the optic fiber secured in the PF^29,90^, followed by electrical stimulation (10 bipolar square-wave current pulses of 0.3 ms duration and variable amplitude (400–800 µA) delivered every 2 s; generated by an isolated pulse stimulator (A-M Systems Model 2100) and gated by an analog stimulus isolator unit (A-M systems Model 2200)). Subsequently, 500 nl of the glutamatergic receptor blocker cocktail were delivered using a Micro-Syringe Pump Controller (World Precision Instruments) and stimulation trials with electrical pulses followed by blue light were repeated. All stimulation trials were repeated at least three times, always preceded and followed by a minimum of 10 s baseline and recovery recording.

### Identification of neurons from extracellular recordings

Neuron activity was used to determine the putative nature of neurons recorded using extracellular tungsten electrodes and high-density silicon probes. Only neurons with a signal-to-noise ratio higher than 1:3, stable action potential waveform during the entire recording and a minimum inter-spike-interval (ISI) of 2 ms were considered as single units. Striatal neurons were categorized based on their basal firing rate, CV, and action potential duration as previously described^23,25,91^. Briefly, putative spiny projection neurons (pSPNs) were defined by their low basal firing rate (<2 Hz), a long peak-to-peak action potential duration (>0.5 ms) and high CV (>1). Putative cholinergic interneurons (pCINs) were defined by a high firing rate (>2 Hz), a long peak-to-peak action potential (>0.5 ms), a long action potential duration (>1.5 ms) and a low CV (<1). Recording and parameters from non-identified extracellular recordings were similar to those from juxtacellularly labeled neurons.

### Electrophysiological data analysis

Spike trains were digitized and converted into a time series of events using in-built Spike2 functions. To determine variations in the firing rate following experimental manipulations, 10-s segments of baseline activity preceding each stimulus were compared to the firing rate during the laser stimulation. Stimulus-induced response of each neuron was expressed as a percentage of change from the mean firing rate of their individual baseline. Neuron responsiveness was determined by computing a time-resolved response to light stimulation as previously described^19^. A modified estimation of the mean instantaneous firing rate (iFR) locked to the laser was obtained for all trials and for each neuron. A cumulative distribution function (CDF) was defined by using each spike train *j* = 1, …, *k* as a sequence of individual events occurring at the time {*t*_*i,j*_}, *I* = 1, …, *n*_*j*_. The number of events up to *t*_*i,j*_ is a strictly increasing function changing at *t*_*i,j*_. The mean density of events across the *k* trials is obtained by dividing the slope of the CDF by the number of trials and represents the trial-averaged firing rate. Then a local linear regression based on *N*_*i,j*_ = 6 × *k* proximal events is used to estimate the iFR at each reference time *t*_*i,j*_ (FR(*t*)). The distribution FR(*t*) of spontaneous activity during baseline (10 s before laser stimulation) was then used to compare the activity during the laser period (8 s) and post-stimulation period (10 s). Percentiles 5 (C_5_) and 95 (C_95_) of FR(*t*) during baseline were selected as thresholds to assess significant responses during laser stimulation. Significance of the response was assessed using cluster-based permutation (*n* = 200 permutations, *P* < 0.05), if the change occurs during laser stimulation and for at least 2 s. If these criteria were not met, then the neuron was considered as non-responding. *Z*-scored data are represented as mean ± 95% coefficient intervals (CI). Significant effects are represented in the figures as horizontal lines. The significance level for all tests set to *P* < 0.05% of changes data are expressed as mean ± SEM.

### In vitro recordings

In vitro whole-cell recordings were performed in adult ChAT::cre+ rats, ChAT-ChR2-YFP mice (Jackson #014546), ChAT-cre mice (Jackson #006410), or WT mice (Jackson #000664) using similar protocols as previously described^45^. Briefly, adult mice or rats (above 3 months age) were deeply anesthetized using isoflurane and then injected with 100 mg/kg ketamine (i.p.). The animals were then transcardially perfused using ice-cold N-methyl d-glucamine (NMDG)-based solution containing (in mM): 103.0 NMDG, 2.5 KCl, 1.2 NaH_2_PO_4_, 30.0 NaHCO_3_, 20.0 HEPES, 25.0 dextrose, 101.0 HCl, 10.0 MgSO_4_, 2.0 Thiourea, 3.0 sodium pyruvate, 12.0 N-acetyl cysteine, 0.5 CaCl_2_ (saturated with 95% O_2_ and 5% CO_2_, pH 7.2–7.4). The rodents were then decapitated and their head immediately transferred to a Petri dish containing oxygenated, ice-cold NMDG-based solution. The brain was extracted and transferred to a Leica vibratome (VT 1200S) containing an oxygenated bath of NMDG-based solution maintained at −4 °C. Sagittal or coronal sections of 250 or 300 μm thickness were cut and transferred to an oxygenated bath of NMDG-based solution maintained at 35 °C for recovery for 5 min. The slices were transferred to a Ringer’s solution bath maintained at 25 °C and allowed to recover for at least an hour. Recordings were performed using glass pipettes (impedance of 3–4 MΩ) containing a regular internal solution composed of (in mM): 130 K-gluconate, 10 KCl, 2 MgCl_2_, 10 HEPES, 4 Na2ATP, 0.4 Na2GTP, pH 7.3.

Alternatively, CsCl-based internal solution (in mM): 125 CsCl, 2 MgCl_2_, 10 HEPES, 4 Na2ATP, 0.4 GTP was used for some voltage-clamp recordings at −70 mV. Pipette internal solution was supplemented with biocytin or Alexa 594 to allow for post hoc identification of the recorded neurons. Axoclamp 700B amplifier (Molecular Devices) and ITC-1600 digitizer (Instrutech) was used to measure membrane currents and voltages. Data acquisition was done using Axograph (www.axographx.com) software. Slices were visualized using Andor Ixon (Andor Technology Ltd., Belfast, Northern Ireland) camera and software.

For in vitro DREADD recordings, ChAT::cre+ rats were injected with AAV-DIO-hM4Di-mCherry virus (500 nl) in the striatum and were allowed to recover for 5–6 weeks before recording. mCherry-positive neurons were recorded and confirmed to be CINs by initial identification of their electrophysiological properties and followed by post hoc immunolabelling for ChAT. Similar recording in mCherry-negative CINs revealed no changes in their firing rate following exposure to CNO (data not shown).

For optogenetic experiments, ChAT-cre mice were injected with an AAV-DIO-mCherry (to label CINs, 200 nl) and an AAV-DIO-ChR2-YFP (to activate transduced CINs, 50 nl). When recording YFP + CINs, optogenetic stimulation produced action potentials. Only CINs neurons not expressing YFP/ChR2 were used for analyses. Optogenetic stimulation consisted of 2–5 ms-duration blue-light pulses delivered using high-power (750 mW, 450 nm) LED. Sweeps were run with an inter-trial interval of 10 s. Each trial was 4 s long with an optogenetic pulse at the 2 s mark.

In vitro carbachol puffs were delivered using a micropipette connected to a Picospritzer (General Valve). Carbamylcholine chloride (Carbachol, 100–250 µM) was dissolved in Ringer’s solution containing 0.5 μM atropine, 10 μM CNQX and d-(−)−2-AP-5. Pressure pulses were applied at 20 psi with a width of 100 ms. Pulses were spaced by at least 3 min to avoid receptor desensitization.

Drugs that were used include: bicuculline (to block GABA-A receptors; 10 µM, Sigma), dihydro-b-erythroidine hydrobromide (DhβE; to block type 2 nicotinic receptors; 1 µM, Tocris), CNQX (to block AMPA receptors; 10 µM, Tocris), d-(−)−AP-5 (to block NMDA receptors; 10 µM, Tocris) and CNO (to activate hM4Di receptors; Sigma, 10 µM). All drugs were dissolved in Ringer’s solution. Data were analyzed using paired two-tailed *t*-test with GraphPad Prism 6 software. Differences were considered to be significant at *P* < 0.05.

### Immunohistochemistry and immunofluorescence

Following behavioral, electrophysiological, or anatomical experiments, rats were humanely euthanized with an overdose of pentobarbital (300 mg/kg, i.p.) and then intracardially perfused with 0.05 M PBS followed by ice-cold 4% paraformaldehyde (PFA) in PBS. Brains were prepared for either coronal or sagittal sections (50 µm) in PBS using a vibrating microtome (VT1200S, Leica). For each experiment, several sections near the injection, drug administration, or recording sites were selected and processed for immunostaining as previously described^19^. For all experiments, injection sites and implantation sites were confirmed by fluorescent microscopy and only those with on-target injections were processed further. Fluorescent images were then overlaid with custom-modified brain templates^92^ for anatomical representation.

Briefly, for juxtacellular experiments, recording site sections were incubated in a blocking solution (10% normal donkey serum in 1% Triton X100 in PBS) for 1 h. Sections were then washed and incubated overnight in an antibody against ChAT and GFP. Following several washes, sections were incubated for 4 h in CY5-conjugated donkey antibody and Alexa-488-conjugated donkey antibody. All sections in the striatum were processed to reveal neurobiotin using CY3-streptavidin solution in PBS-0.03% Triton. Sections containing CY3-positive neurons were incubated with antibodies against PV, chicken ovalbumin upstream promoter transcription factor interaction protein 2 (Ctip2) or ChAT, based on their relative firing properties, presence of spines and soma size. Following overnight incubation at 4 °C and several washes, sections were incubated with their respective fluorophore-conjugated secondary antibody (see Table 3). Images of streptavidin-positive neurons were captured using a confocal microscope (LSM-510, Zeiss). If the neurochemical nature of the neurons was not revealed following the first immunostaining, sections were re-incubated with different antibody (against PV, Ctip2 or ChAT). Neurons that were negative for all three antibodies were considered as others (non-PV GABAergic interneurons) and not considered further in this study.

**Table 3:** Antibodies list.

Target	Raised in	Cat. number	Dilution	Company
Primary antibodies				
ChAT	Goat	AB144P	1/500	Millipore Sigma
GFP-488	Rabbit	A21311	1/1000	Invitrogen
PV	Guinea Pig	195004	1/500	Synaptic System
Ctip2	Rabbit	AB28448	1/500	Abcam
HA	Rabbit	3724	1/1000	Cell Signaling
mCherry	Rabbit	AB167453	1/1000	Abcam
pser240-244	Rabbit	2215	1/250	Cell Signaling
GFP	Rat	04404	1/1000	Nacalai Tesque
Secondary antibodies				
Anti-Goat CY5	Donkey	705-175-147	1/250	Jackson Immunoresearch
Anti-Goat CY3	Donkey	705-165-147	1/500	Jackson Immunoresearch
Anti-Rabbit 488	Donkey	711-545-152	1/500	Jackson Immunoresearch
Anti-Rabbit CY3	Donkey	711-165-152	1/500	Jackson Immunoresearch
Anti-rat biotinylated	Rabbit	BA4000	1/500	Vector Labs
Anti-Goat IgG	Donkey	705-005-147	1/100	Jackson Immunoresearch
Goat-PAP	Goat	123-005-024	1/200	Jackson Immunoresearch

For the histology of brains of animals from behavioral experiments, sections within the striatum and the PPN/LDT were processed for mCherry or HA (Human Influenza) and ChAT with their respective fluorophore-conjugated secondary antibodies. Images of the cannula implantation site and PPN/LDT were acquired using a confocal microscope. Due to the weak labeling of axons from DREADD expressing neurons, a cholinergic neuron expressing mCherry and FG was considered as projecting to the striatum and responsive to CNO administration.

### Monosynaptic modified-rabies labeling and input neuron analysis

Two floxed viruses were co-injected into the striatum to induce the expression of a TVA receptor (AAV-FLEX-TVA-mCherry) and G glycoprotein (AAV-FLEX-G) in direct and indirect pathway neurons (subsequent to the injection of Cav2-Cre in the SNR and GPE). In addition, the same helper viruses were injected in the striatum of ChAT::Cre rats to target CINs. Two weeks later, a G-deleted pseudotyped rabies virus (SAD∆G-eGFP) was injected into the striatum to infect neurons expressing the TVA receptor (starter neurons). Neurons also expressing the G glycoprotein allowed the transsynaptic transport of the pseudotyped rabies virus, thus labeling those neurons that have monosynaptic connections with Cre-expressing striatal neurons (input neurons). Seven-to-ten days later, the rats were perfused-fixed and their brains analyzed.

Following immunostaining of SAD∆G-YFP and TVA (mCherry), quantification of starter neurons (mCherry+/GFP+) and SAD∆G-YFP neurons in the PPN/LDT was performed. For starter neurons quantification, low-magnification (×10), high-resolution (1024 × 1080) confocal images of the injection site in the striatum were acquired. AAV-TVA (mCherry) diffusion volume within the striatum was then determined using in-build freehand tools (ImageJ). In three striatal sections, spaced by 300 µm and containing the injection site, a total of 18 sites of 200 µm^2^ were randomly selected and the total number of neurons overlapping mCherry and YFP signal were counted offline. Using the diffusion area and the number of starter neurons counted in each animal, we estimated the approximate number of starter neurons per animal. For PPN/LDT inputs neurons, all sections containing PPN or LDT that were labeled for ChAT immunostaining and YFP signal were counted. PPN and LDT (defined by ChAT staining) were scanned at high-magnification (×20), high resolution (1024 × 1080) using mosaic confocal in-build tools (Fluoview FV1260, Olympus). Mosaic files were then transferred to ImageJ software, the contrast was adjusted and borders of the PPN/LDT defined using freehand tools. Positions of the YFP-positive neurons were recorded using multi-point in-build tools and digitized on a representative sagittal section of the PPN/LDT. Counted neurons within the PPN/LDT borders were then expressed as a ratio of inputs neurons within the PPN/LDT per 1000 starter neurons.

### PSer240-244 detection and fluorescence analysis

ChAT::cre+ rats injected in the PPN with AAV-EF1a-DIO-ChR2-YFP (*n* = 5) or AAV-EF1a-DIO-YFP (*n* = 5) were deeply anesthetized with 4% v/v isoflurane (Schering-Plough) in O_2_, maintained with urethane (1.3 g/kg, i.p.; ethyl carbamate, Sigma, Poole, UK) and placed on a stereotaxic frame. A flat-cut optic fiber (200 µm, 0.39NA, Thorlabs) was inserted in the DLS. Animals received 10 stimulation trains (10 Hz, 50 ms pulses, 8 s duration, 30 s inter-stimulation interval) and were transcardially perfused 30 min following stimulations. Following slicing, free-floating brain sections from around the stimulation site were collected. Sections were blocked as described above and then incubated overnight for immunostaining of ChAT and the double phosphorylated S6 ribosomal protein (pSer^240–244^-S6p). Sections were then rinsed several times and incubated with their respective fluorophore-conjugated secondary antibodies.

High-resolution images of ChAT+ neurons in the striatum surrounded by YFP+ projections were acquired (×40, in oil, resolution 1024 × 1080) and examined under confocal microscopy (Fluoview FV1200, Olympus) using 560 and 650 nm lasers. Three regions of interest (ROI) were defined using ChAT staining, ROI1: ChAT-immunoreactive area (neuron surface excluding the nucleus), ROI2 and ROI3: background signal in the vicinity of the soma. 32-bits pictures were converted to grayscale images using ImageJ (color tools) and the mean gray value (fluorescence intensity: FI) of each ROI was obtained for the pSer^240-244^ channel using ImageJ (mean gray value). For every picture, background fluorescence intensity, ROI2(FI) and ROI3(FI) were compared and averaged (FI = x̅ (ROI2(FI)/ROI3(FI)), then soma FI was compared to the background in order to obtain ΔFi (ROI1(FI)- x̅ (Roi2(FI)/Roi3(FI)). We used a two-tailed *t*-test for two-group comparisons (ChR2-YFP and YFP) and compared background FI, soma FI and ΔFi^24^. Images scanning, gray-scaling, and analyses were done using blind experimenters. Two blinded experimenters performed the quantification of pSer240-244 fluorescence. Background fluorescence and normalized somatic fluorescence were analyzed using one-way ANOVA. The significance level for all tests was set to *P* < 0.05.

### Electron microscopy

Sections from ChAT::cre+ rats injected in the brainstem with AAV-DIO-GFP were collected as described above. Sections were incubated in a cryoprotectant solution (0.05 M phosphate buffer, 25% sucrose, 10% glycerol) overnight, then freeze-thawed in order to increase penetration of the reagents. Sections were double-immunolabeled to reveal postsynaptic intrastriatal CINs using an anti-ChAT antibody and presynaptic YFP-containing axons using a biotinylated antibody against GFP. Sections were blocked in 10% normal rabbit serum (NRS, Vector) in PBS for 2 h prior to incubation of primary antibodies. All sections were incubated for 24 h at room temperature in a rat antibody against GFP (1:1000; Nacalai Tesque Inc.) followed by an incubation in a rabbit anti-rat biotinylated antibody (1:500; Vector Labs) overnight at 4 °C. After washing thoroughly, sections were incubated in an avidin–biotin–peroxidase complex (ABC Elite, 1:100; Vector) for 3–4 h at room temperature. The sections were incubated in Tris-buffer (0.5 M, pH 8; TB containing 0.025% DAB solution [DAB wt/vol, Sigma] and 0.5% nickel ammonium sulfate [wt/vol, Sigma] for 20 min). The reaction was initiated by addition of H_2_O_2_ to a final concentration of 0.01%. To label cholinergic structures, all sections were incubated in a goat antibody against ChAT (1:500 in PBS NRS 1%; Millipore) for 24 h at room temperature. Sections were then incubated in rabbit anti-goat IgG (1:100 in PBS NRS 1%; Jackson Immunoresearch) for 4 h at room temperature, followed by incubation in goat peroxidase–anti-peroxidase (1:200 in PBS; Jackson Immunoresearch) for 2 h at room temperature. The sections were washed in PBS followed by washes in 0.1 M phosphate buffer (PB), pH 6.0. Sections were pre-incubated in PB containing TMB as the chromogen. The reaction was initiated by adding glucose oxidase (Sigma G6891) and stopped after 6–8 min with 0.1 M PB. The reaction was stabilized with DAB and cobalt chloride and washed thoroughly before being processed for electron microscopy.

The sections were postfixed in osmium tetroxide (1% in PB, Oxkem, Oxford, UK) for 20 min. The sections were dehydrated and contrasted with 1% uranyl acetate and left overnight in resin (Durcupan, ACM, Fluka, UK) before being mounted on slides and cured at 60 °C for at least 48 h. All sections were examined in the light microscope and areas of interest were cut from the slide, embedded in resin blocks and trimmed with a razor blade. Serial sections of 50 nm thick were cut using an ultramicrotome (Leica EM UC6), collected in copper grids and lead-stained to be examined on a Philips CM10 electron microscope.

Striatal cholinergic axons of PPN origin were identified by the presence of a black dense material produced by the deposition of the DAB-nickel in axons and terminals. Intrastriatal CINs and their processes could be identified by the presence of TMB crystals within dendrites and cell bodies.

### Goal-directed test

All animals used for behavioral experiments were handled, and dummy cannula replaced, on a daily basis to avoid stress-related behavior. Both ChAT::Cre and WT male Long Evans rats were used for behavioral testing. Experiments were performed during the dark phase of the light cycle and the room was illuminated with red light to maintain behavioral activity. Four weeks following virus injection and cannula implantation, rats were randomly assigned to an operant box containing one lever (at the right or the left side of the box, randomly attributed) and at the opposite side of a food pellet dispenser. A small LED was placed above the magazine and the box was illuminated by a house light (MED-008-D1, Med Associates). Before training, rats were exposed to sugar pellets (45 mg, Bio-Serv, Frenchtown, NJ) in their home cage and then in the operant box magazine. Following two days of habituation, animals were manually shaped for lever pressing until completion of 80 presses within 40 min on a fixed-ratio-1 (FR1; one press, one reward) schedule. House light was kept on and the lever was continuously extended during testing. Training occurred at the same time of the day, 7 days a week.

### Outcome devaluation test

Following the completion of a successful FR1 session, rats were trained on a random-ratio schedule (RR5; on average one reinforcer every 5 lever presses, minimum 1 press, maximum 9 presses) for 30 min during 4 days. Animals received CNO (1 µM) delivery through the bilateral cannulas 30 min before daily training. During CNO delivery, animals were gently restrained, dummy cannula removed, and injectors (30 gauges) were lowered. Injections were delivered with a 30 gauge indwelling cannula connected to a high-precision pump (New Pump 11 Elite, Harvard Bioscience, Canada). A total of 250 nl of CNO was infused over 5 min in each hemisphere. Following infusion, the cannula was kept in place for 2 min and the animal returned to the home cage.

The outcome devaluation test occurred in the following two days. Animals were exposed 30 min before the behavioral test to sugar pellets (devalued session) or to lab chow pellets (valued session). On the valued session, rats had ad libitum access to the home-cage outcome (lab chow pellet) while on the devalued session, rats had ad libitum access to sugar pellets. Valued and devalued sessions were randomized between animals and consisted of a non-reinforced RR5 session of 15 min.

### Habit learning test

Following the first outcome devaluation, animals were re-trained on a FR1 schedule until completion of 80 presses within 30-min sessions (in order to avoid CNO effects). Following successful FR1 completion, animals were trained on a progressive RI schedule consisting of 2 days of RI15 (one reinforcer following the first press after an average of 15 s, minimum time 1 s, maximum time 30 s), 3 days of RI30 (average of 30 s, minimum time 1 s, maximum time 60 s) and 3 days of RI60 (average of 60 s, minimum time 1 s, maximum time 90 s). During sessions of RI training, animals received CNO 30 min before the behavioral test. Following the completion of the RI schedule, rats were tested on two consecutive outcome devaluation tests (as previously described), consisting of two non-reinforced RI60 sessions of 15 min each. During all behavioral sessions, head entries, timestamps of reward delivery, and timestamps of lever presses were recorded.

Following the last outcome devaluation session, animals were tested for locomotion for 2 days. Animals received CNO delivery through the cannula (days were randomly distributed). 30 min following infusion, animals were placed in a 50 cm × 50 cm open field for 30 min. Horizontal activity and time spent in the center (design of a central square of 20 cm × 20 cm) were detected using AnyMaze software (StoeltingCo) and an infrared camera (Logitech). Drugs and animal orders were randomized.

Following the completion of all behavioral experiments, animals received injections of fluorogold through the cannula (300 nl) to retrogradely label projecting neurons in the PPN and confirm cannula location and diffusion. One week after fluorogold injections, animals were perfused and their brains processed as previously described. Animals that did not complete all experiments (training, devaluation, and locomotion) or animals that had the cannula misplaced or did not show any fluorogold labeling (cannula blocked) were removed from all analysis (WT: *n* = 4; PPN: *n* = 1; DMS: *n* = 2; LDT: *n* = 4).

### Locomotor activity in open field

To assess changes in motor activity potentially associated with the chemogenetic inhibition of PPN cholinergic neurons, ChAT::Cre+ rats were transduced with hM4Di and evaluated in the open field after the systemic injection of CNO (1 mg/kg). The open field consisted of a square arena (82 cm × 82 cm × 46 cm) with dark gray walls. The USB connected camera (Genius USA) was set ~120 cm above from the floor of the arena. The central area (40 cm × 40 cm) was set in the middle of the arena. The peripheral area was delineated from the boundary of the central area to the wall of the arena. Tracking the animal and assessing the total traveled distance of the task data were done using ANY-maze (StoeltingCo). On day 1, all the animals received an IP injection of saline and explored the arena freely for 5 min. On days 2 and 3, animals were segregated into two groups randomly. On day 2, group 1 received a saline injection while group 2 received CNO injection 40 min prior to open field testing. On day 3, group 1 received a CNO injection, while group 2 received a saline injection 40 min prior to open field testing. The total time in the open field arena each day was 25 min. The data for the first five minutes were excluded from the analysis.

### Sugar consumption

Following locomotor activity testing, sugar consumption was tested for 4 days. Animals received CNO (2 days) and vehicle (2 days) delivery through the cannula (days were randomly distributed). 30 min following infusion, animals were returned to their home cage and were given access to sugar pellets for 10 min (10 g). Following the completion of the 10 min, the sugar cup was weighted, and the difference was recorded.

### RR task

Both male and female ChAT::Cre+ Long Evans rats and WT were used for the RR test. All animals received an injection of AAV-Flex-hM4Di-mCherry in the rostral PPN. Experiments were performed during the dark phase of the light cycle and the room was illuminated with red light to maintain behavioral activity. Four weeks following virus injection, we implanted cannulas in the DLS. Cannulas were used for the local administration of CNO (250 nl, bilateral, 1.5 µM), which occurred 30 min before testing. Rats were randomly assigned to an operant box containing two levers (randomly attributed) and in the middle a food pellet dispenser. A small LED was placed above the magazine and the box was illuminated by a house light (MED-008-D1, Med Associates) located at the top of the opposing wall. Before training, rats were exposed to sugar pellets (45 mg, Bio-Serv, Frenchtown, NJ) in their home cage and then in the operant box magazine. Following two days of habituation, animals were manually shaped for lever pressing until completion of 80 presses within 40 min on a fixed-ratio-1 (FR1; one press, one reward) schedule. House light was kept on and the lever was continuously extended during testing. Training occurred at the same time of the day, 7 days a week. When rats pressed on the “correct” lever, the light above the food pellet dispenser illuminated and a food pellet was dispensed, then the house light was extinguished for 10 s (inter-trial interval) signaling the end of the trial. If rats pressed the “incorrect” lever at any point, the house light was extinguished for 10 s signaling the end of the trial. All lever presses and magazine entries were recorded and used to form a variety of behavioral measures. Each session lasted for 40 min. Rats were advanced through a variety of RR reinforcement schedules. For RR5 (5 consecutive days), correct presses necessary during each trial was set between 1 and 9 with a mean of 5. For RR10 (3 consecutive days), correct presses necessary during each trial was set between 1 and 19, with a mean of 10. The following day after the 3rd session of RR10, animals were tested for extinction (‘ext’), for 3 consecutive days with each session lasting 40 min. During extinction, the stimuli were presented similar to the RR10 schedule but no reward was delivered.

### Statistical analyses

No statistical methods were used to predetermine sample size, but all sample sizes were similar to previously reported behavior, cellular or in vivo electrophysiological studies^8,19,20,45,70^. Animals with injections or implantation sites out of target were excluded using pre-established criteria.

The normality of each data set was assessed using the Shapiro–Wilk test. For behavioral experiments, drugs, assigned boxes, testing order, and devaluation order were randomized using unbiased methods. The number of lever presses, lever-press rate, and locomotion data were analyzed using repeated-measures ANOVA, using Tukey or Bonferroni post hoc analyses when appropriate. Lever presses were normalized using: (lever press for Valued or Devalued states/total lever presses Valued + Devalued states). For outcome devaluation, data were analyzed using two-way ANOVA comparing the number of presses and normalized number of presses during valued and devalued periods for each condition (RR, RI). We examined the strength of the outcome devaluation by testing the devaluation index [(valued lever presses—devalued lever press)/total lever presses] in the goal-directed (RR) and habitual learning (RI) contexts. Devaluation index data for each group were analyzed using paired two-tailed *t*-tests. Note that the statistical main effect of group is not reported as all group means equal 0.5 due to the normalization. For electrophysiological and anatomical normally distributed data, ANOVAs or *t*-tests (paired or unpaired) were performed; for data that were not normally distributed or for small samples (i.e. monosynaptic tracing), we used Kruskal–Wallis rank-sum test (one-way ANOVA on ranks) and post hoc analysis using the Wilcoxon rank-sum test (Mann–Whitney *U* test). Data analysis was performed using SPSS (IBM) or the statistical toolbox from Matlab. All data are reported as mean ± SEM.

### Contact for reagent and resource sharing

Further details and specific requests for resources, data, code, materials or protocols used in this manuscript should be directed to and will be fulfilled by the corresponding author.

### Reporting summary

Further information on research design is available in the Nature Research Reporting Summary linked to this article.

## Supplementary information

Supplementary Information

Peer Review

Reporting Summary

## Data Availability

In vivo electrophysiology data, in vitro electrophysiology, and behavior data are available under reasonable request to the corresponding author.

## Code availability

All code used to collect and analyze data are available under reasonable request to the corresponding author.
