# Supplementary material for: Cholinergic midbrain afferents modulate striatal circuits and shape encoding of action strategies
Source: Nat Commun. 2020 Apr 8;11:1739. doi: 10.1038/s41467-020-15514-3 (PMC7142106; doi:10.1038/s41467-020-15514-3)
Supplement: Supplementary file 1 — Supplementary Information [file 41467_2020_15514_MOESM1_ESM.pdf]

Supplementary Information

Cholinergic Midbrain Afferents Modulate Striatal Circuits and Shape Encoding of Action Strategies

Dautan et al.

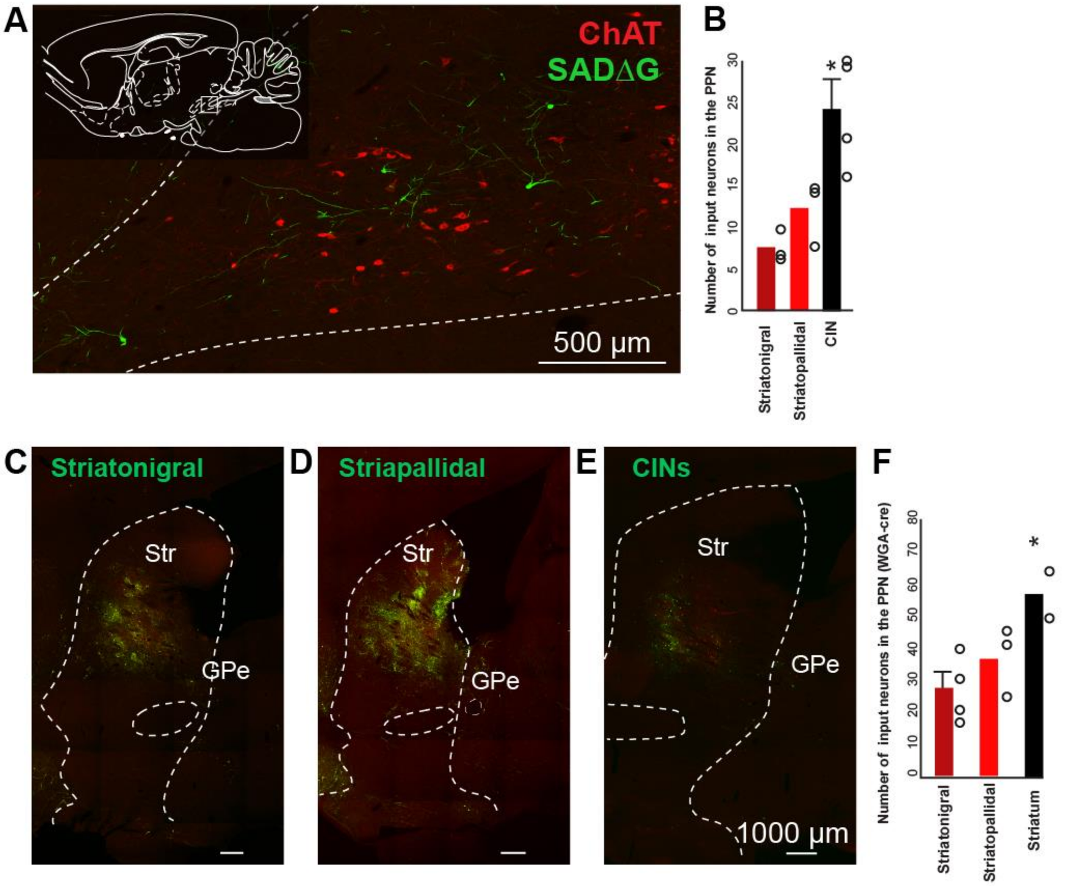

Supplementary Figure 1: Low magnification images of tracing experiments. Related to Figure 1.

(A) Low magnification image of input neurons in the PPN. Square in the inset outline shows the location within the PPN.

(B) Quantification of total input neurons in the PPN and LDT (each circle represents one rat, obtained from 3 rats in striatonigral and striatopallidal labeling, and 4 rats in CINs). * $P < 0.05$ (direct SPNs: 7.33 ± 0.88; indirect SPNs: 12 ± 2; CINs: 24 ± 3.34, Kruskal-Wallis test H(2)=7.436, $P = 0.0243$, *post hoc* Tukey test: $P_{iSPNs\text{-}dSPNs} = 0.498$, $P_{iSPNs\text{-}CINs} = 0.033$, $P_{dSPNs\text{-}CINs} = 0.007$).

(C-E) Low magnification images showing of the extent of viral diffusion labeling of starter neurons. Area of transduction in mm^2: direct SPNs: 1.04 ± 0.0082; indirect SPNs: 1.36 ±0.01; CINs: 1.62 ± 0.0069, Kruskal-Wallis test H(2) = 2.091, $P = 0.3515$. Number of starter neurons: direct SPNs: 357.55 ± 37.43; indirect SPNs: 367.06 ± 22.57; CINs: 96.32 ±7.68.

(F) Normalized quantification of input neurons in the PPN and LDT using WGA-Cre instead of Cav2 (n = 4 rats in striatonigral, n = 3 striatopallidal, n = 2 all striatal cells). Note that this tracer is expected to diffuse transsynaptically across striatal neurons and therefore overestimate the number of input neurons. * $P < 0.05$ (Kruskal-Wallis test H(2)=7.395, $P = 0.0248$, *post hoc* Tukey test: $P_{dSPNs\text{-}all} = 0.026$; $P_{iSPNs\text{-}all} = 0.021$, $P_{iSPNs\text{-}dSPNs} = 0.710$).

All experiments have been replicated at least 3 times.

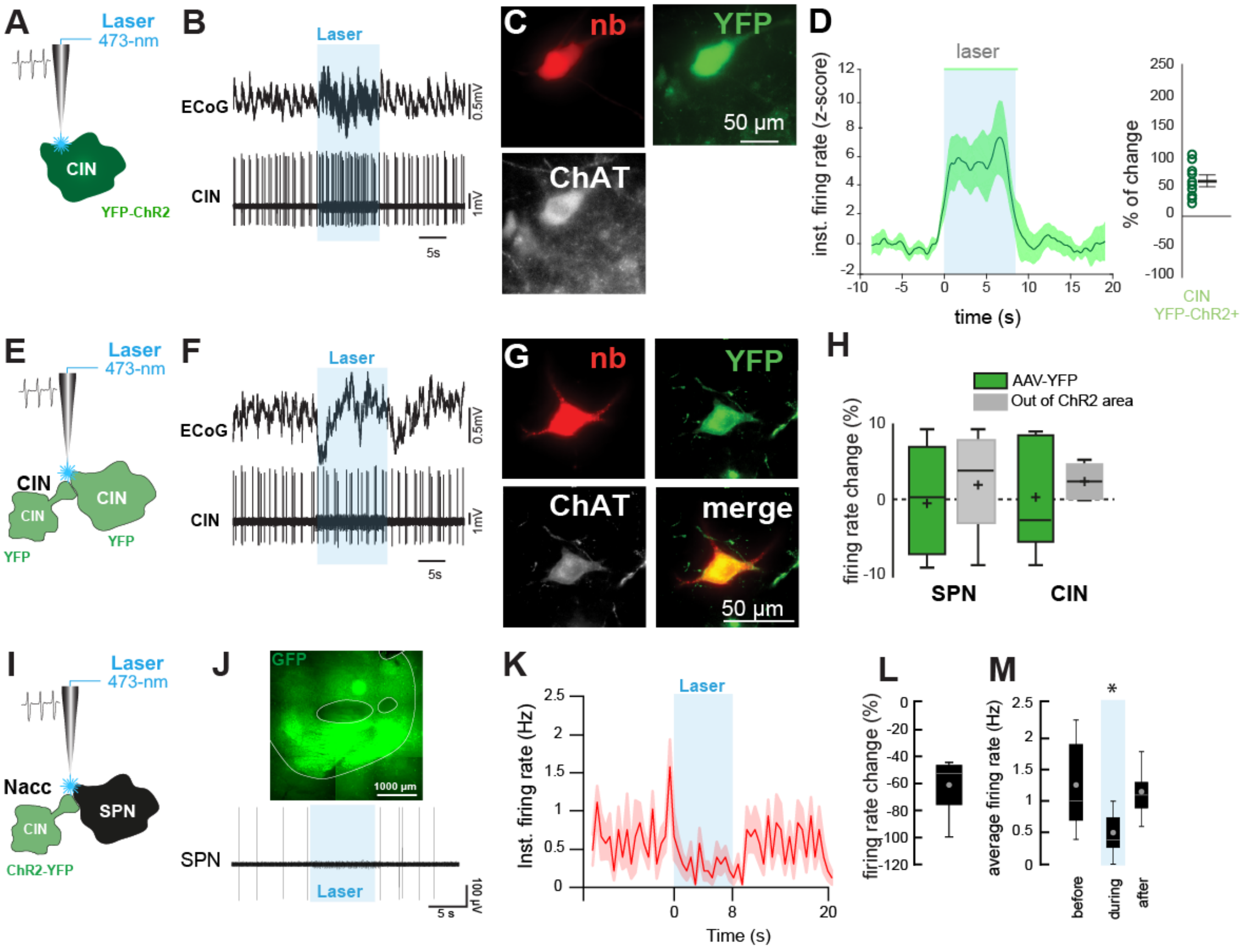

Supplementary Figure 2. Controls for optogenetic experiments. Related to Figures 2 and 3.

(A) AAV-DIO-EF1a-eYFP-ChR2 or (E) AAV-DIO-eYFP injected in the striatum of ChAT::cre+ rats. (B) YFP-ChR2-positive CINs (n = 11) or (F) YFP-positive CINs (n = 11) were recorded with a glass micropipette and blue light was delivered to the recorded neurons. (C) YFP-ChR2-positive or (G) YFP-positive somata were found around the recording site and were revealed by ChAT immunostaining. (D) The normalized instantaneous firing rate of all CINs expressing ChR2-YFP show fast activation dynamics during stimulation (cluster-based permutation test, $P < 0.001$). A significant increase of the firing rate was observed for animals injected with AAV-DIO-ChR2-YFP (firing rate: before laser stimulation 3.9 ± 1.28 Hz; during laser stimulation 6.14 ± 1.43 Hz; after laser stimulation 4.046 ± 1.32 Hz, paired 2-tail t-test before vs. after: t(10) = -3.588, $P = 0.005$) but not for animals injected with AAV-DIO-YFP (firing rate: before laser stimulation 4.92 ± 2.19 Hz, during laser stimulation 4.95 ± 2.20 Hz, after laser stimulation 4.94 ± 2.15 Hz, n = 11 neurons in 4 animals; paired 2-tail t-test before vs. after: t(10) = -0.587, $P = 0.570$; data not shown). (H) No change in the firing rate of striatal neurons was detected in animals transduced with YFP or when the recorded neurons were outside of the ChR2-transduced area (n=17 neurons). (I-M) SPNs recorded in the nucleus accumbens (n = 11) showed a decrease in their firing rate during CINs stimulation, like the inhibition reported in the dorsal striatum. However, the inhibition was shorter in duration (unpaired 2-tail t-test).

Individual data points and mean ± SEM are shown. * $P < 0.05$. Box plots are represented using the mean, median, minimum and maximum value first quartile and third quartile. All experiments have been replicated at least 3 times.

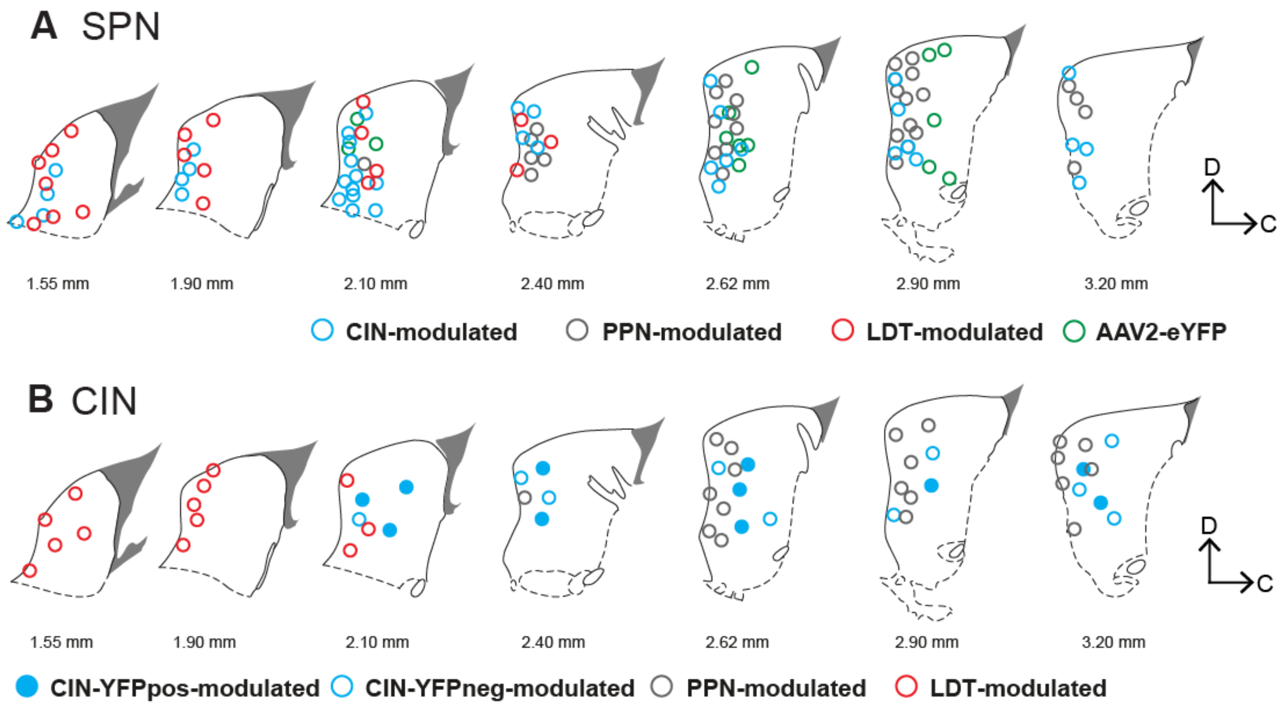

Supplementary Figure 3. Localization of recorded neurons in the striatum. Related to Figures 2 and 3.

Localization of recorded and subsequently labeled neurons in the striatum confirmed as SPNs (A) or CINs (B), following CINs stimulation (blue circles), PPN stimulation (gray circles), LDT stimulation (red circles) or control animals (green circles). Neurons are represented on medial to lateral sagittal planes of the striatum; approximate medio-lateral coordinates from Bregma are provided.

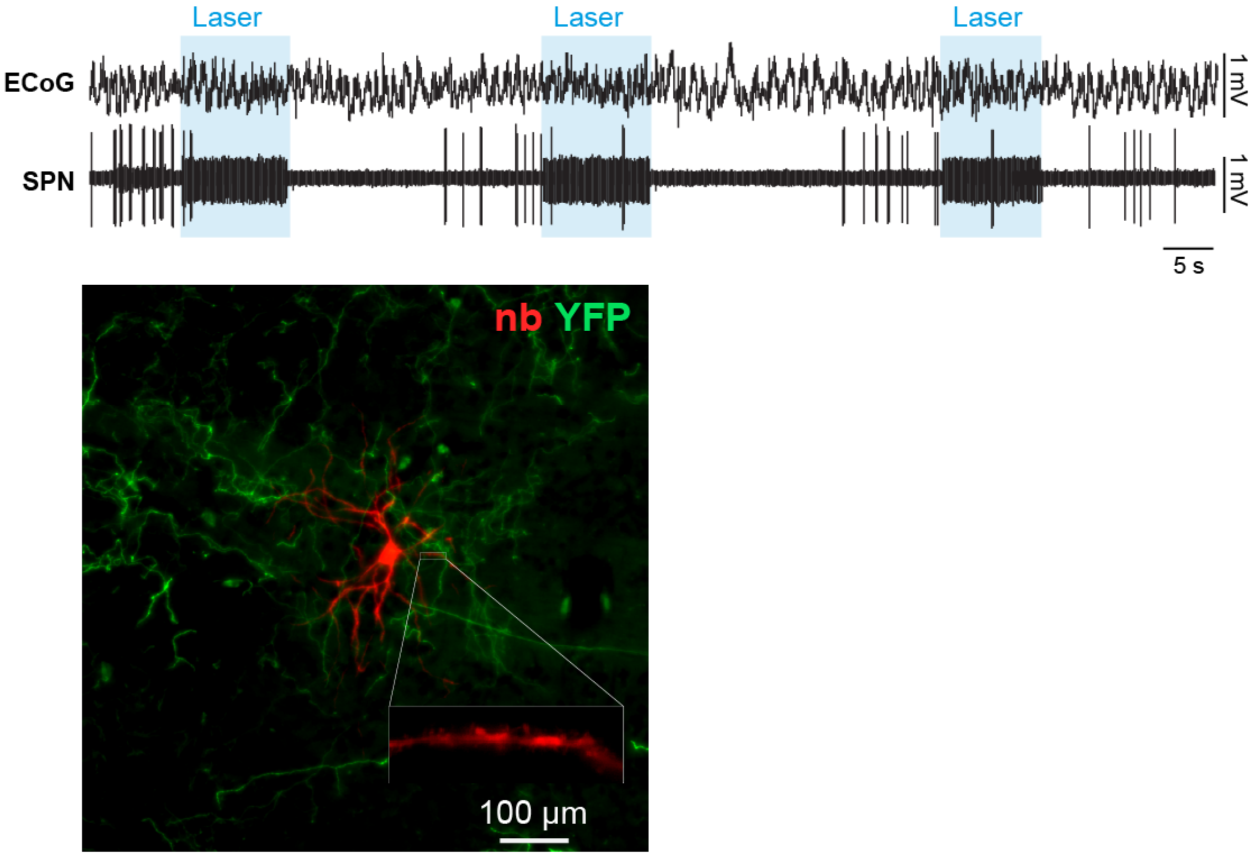

Supplementary Figure 4: Extended recording of a SPN. Related to Figure 2.

Individual identified SPN neuron activity during 3 subsequent stimulation trials of PPN cholinergic axons in the striatum. This neuron was surrounded by YFP-positive axons. All experiments have been replicated at least 3 times.

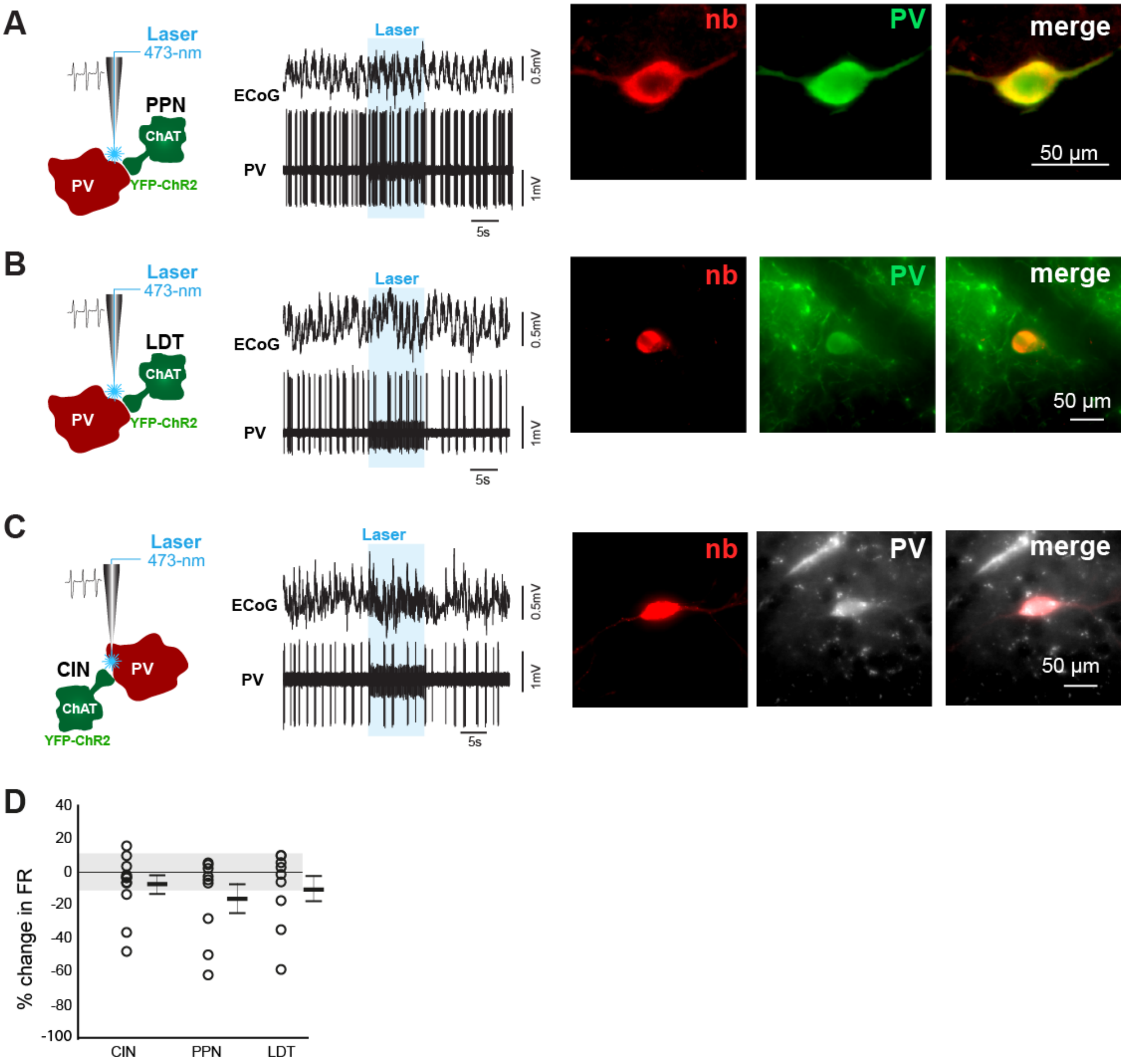

Supplementary Figure 5. Optogenetic activation of cholinergic inputs does not modulate parvalbumin-expressing (PV) interneuron firing rates. Related to Figures 2 and 3.

(A) AAV-DIO-EF1a-eYFP-ChR2 injected in the PPN of ChAT::cre+ rats. PV interneurons in the striatum were recorded with a glass micropipette and the cholinergic axons were stimulated optogenetically (n = 8 neurons). Individual recordings were obtained and the cells were subsequently revealed with neurobiotin and neurochemically characterized.

(B) Same experimental design to assess modulation of striatal PV interneurons by LDT cholinergic axons (n = 9 neurons).

(C) Same experimental design to assess modulation of striatal PV interneurons by cholinergic axons arising from local CINs in the dorsal striatum (n = 11 neurons).

(D) No differences in the firing activity of PV-positive neurons recorded across groups were observed (PPN: 8.55 ± 2.66 Hz; LDT: 7.16 ± 1.42 Hz; CIN: 8.43 ± 2.47 Hz; one-way ANOVA, $F_{(2,25)} = 0.11$, $P = 0.8968$; data not shown). Following photostimulation of cholinergic axons no differences were found in the changes in the firing rates (D, CIN: 1.084 ± 18.58 %, PPN: -1.38 ± 15.91 %, LDT: -3.14 ± 11.56 %; one-way ANOVA: $F_{(2,25)} = 0.70$, $P = 0.5$).

Individual data points and mean ± SEM are shown.

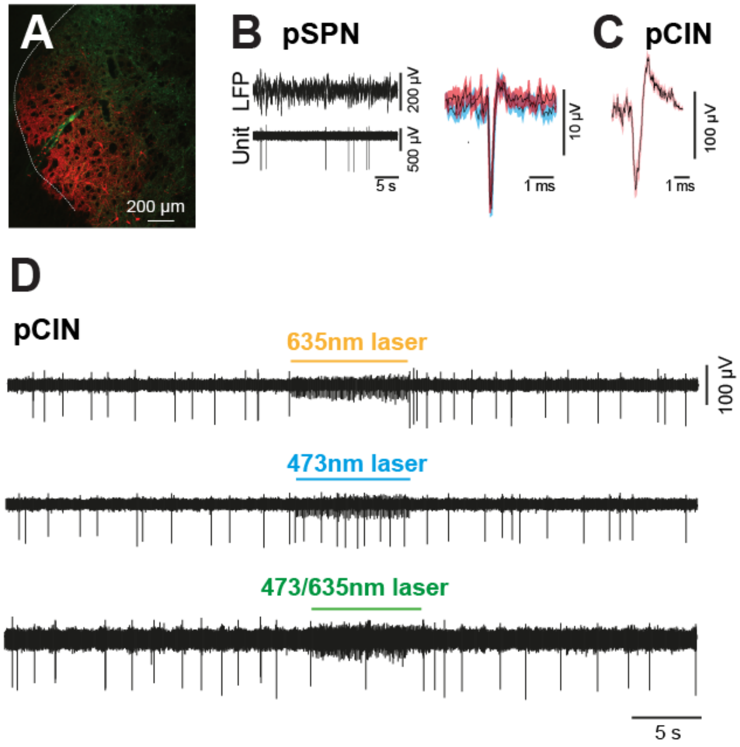

Supplementary Figure 6: *In vivo* extracellular recordings in the striatum. Related to Figure 5.

(A) Low-magnification fluorescent image of an overlapping area of striatum containing cholinergic PPN/LDT axons (YFP) and CINs (mCherry).

(B, C) Representative examples of a putative SPN (B, pSPN) or a putative CIN (C, pCIN) recorded using 16-channels silicon probes. pSPN and pCIN firing activity in multi-contact recordings was similar to that observed in juxtacellularly recorded neurons that were subsequently identified as SPNs or CINs (the trace in B belongs to a pSPN). The average waveform of a pSPN in B during baseline conditions (red) and light stimulation (blue) suggests that there is no effect of the laser stimulation on spike properties in multi-contact extracellular recordings.

(D) Response of a representative pCIN expressing NphR3.0-mCherry following yellow (635 nm), blue (475 nm) or combined yellow/blue laser stimulation. pCINs were inhibited by yellow light (top trace) and excited by blue light (middle trace). Group data shown in Figure 5.

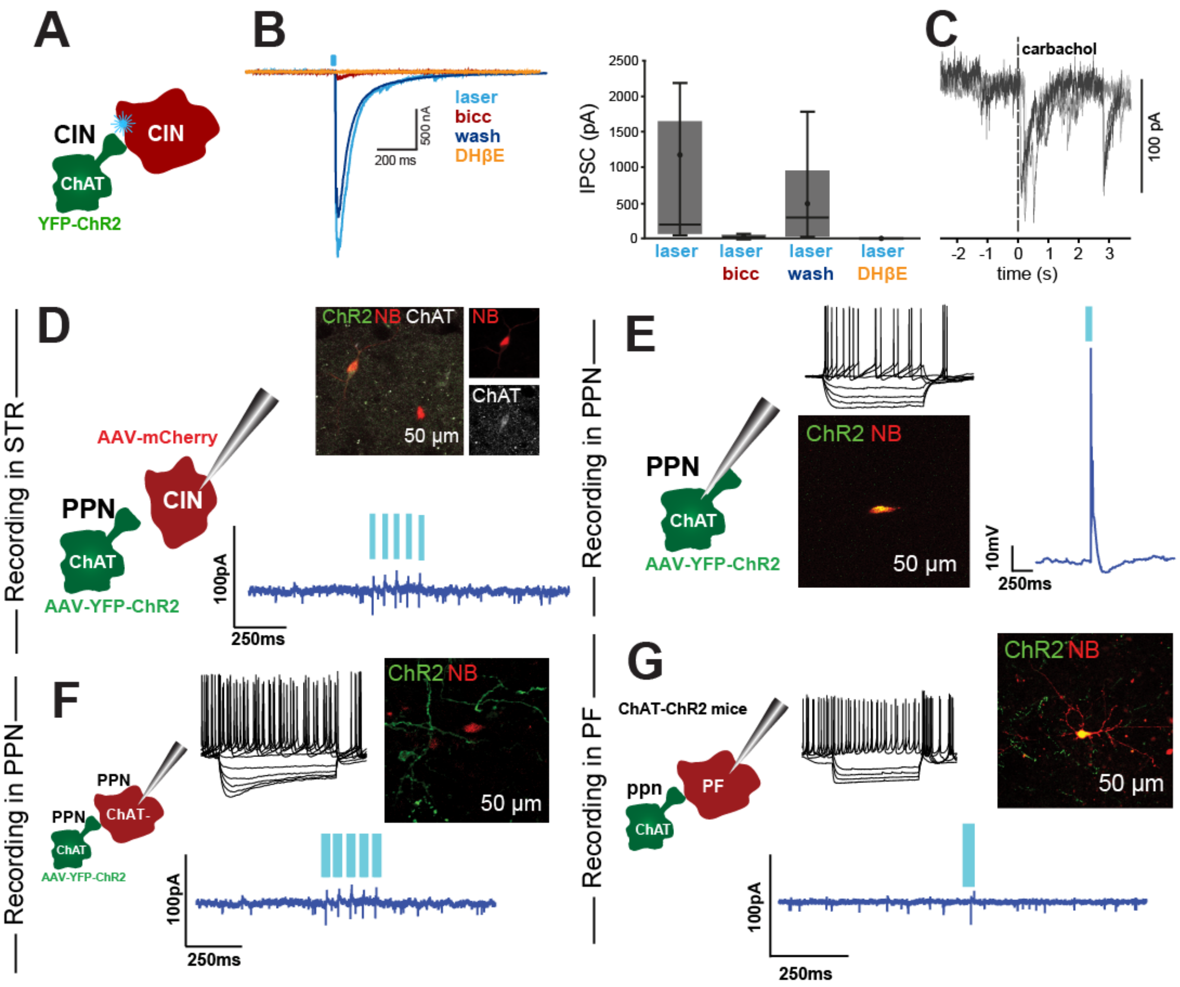

Supplementary Figure 7. *Ex vivo* recordings of CINs. Related to Figure 5.

(A) Schematic of the in vitro optogenetic experiment design for whole-cell recordings of CINs in ChAT::Cre mice transduced with ChR2 in the striatum.

(B) Whole-cell voltage clamp (Vh=-70 mV) recording of identified, non-transduced CINs following optogenetic stimulation of ChR2 (5ms, 450nm) showing consistently IPSCs driven by neighboring CINs axons (n=9 neurons). IPSCs were strongly reduced by bicuculline (n=6 neurons) or a nicotinic receptor blocker (DhβE, n=4 neurons). Box plots are represented using the mean, average, minimum and maximum value first quartile and third quartile.

(C) Whole-cell voltage clamp recording (Vh=-70 mV) of an identified CIN (n=4/11 responding neurons) receiving a local puff of carbachol (100-250 µM, 1 puff/3m).

(D-E) A series of experiments were aimed to characterize the postsynaptic responses of CINs to PPN/LDT cholinergic axon stimulation in ex vivo recordings. Experiments were carried out in both ChAT::Cre rats and mice using a variety of opsins, AAV serotypes, transduction times, laser stimulation parameters and recording solutions, but no responses were obtained in CINs (D). Similar conditions were used for some of the most well-known PPN targets, including the local PPN neurons (F) and the parafascicular nucleus (PF, G). Despite PPN cell bodies displaying spiking activity following laser stimulation (E), no postsynaptic responses were identified in any of their targets.

(D) Whole-cell patch clamp recording of CINs following stimulation of PPN inputs. The average voltage clamp traces (Vh=-70mV) show no postsynaptic responses to a 5 pulses (20Hz) train of optogenetic stimulation.

(E) Whole-cell patch clamp recording of PPN cholinergic neurons visually confirmed as expressing ChR2-YFP. Laser stimulation was always inducing spiking activity following short (2ms) blue pulses, with short latency.

(F) Whole-cell patch clamp recording of ChR2-YFP negative neurons of the PPN surrounded by cholinergic terminals. The average voltage clamp traces (Vh=-70mV) show no postsynaptic responses to a 5-pulses train (20Hz) of optogenetic stimulation.

(G) Whole-cell patch clamp recording of unidentified PF neurons surrounded by ChR2-YFP PPN cholinergic terminals. The average voltage clamp traces (Vh=-70mV) show no postsynaptic responses to optogenetic stimulation.

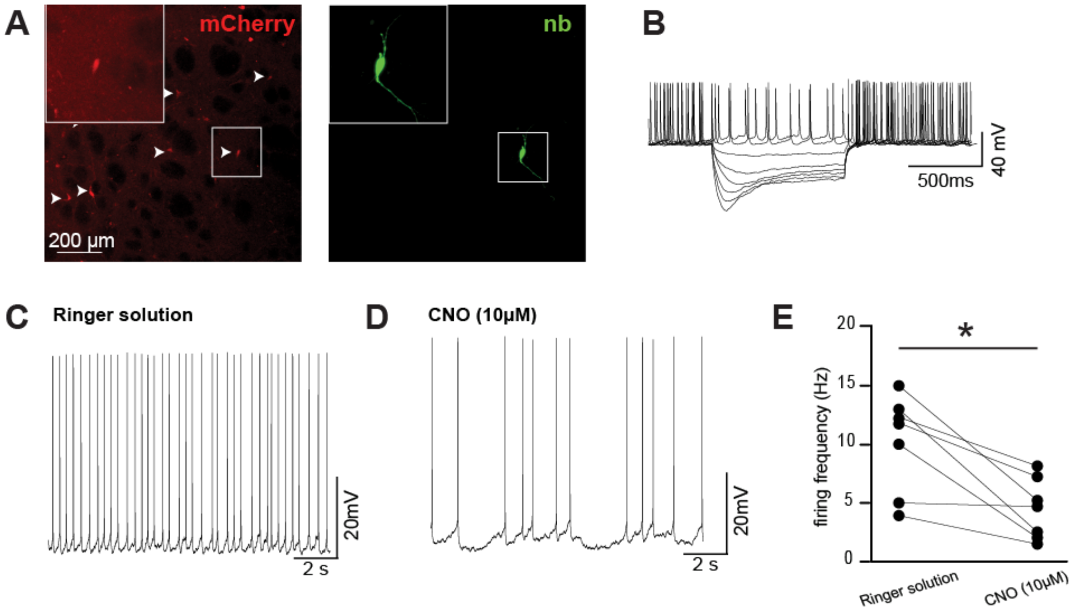

Supplementary Figure 8: Effect of CNO administration on *in vitro* firing activity of CINs. Related to Figures 6 and 7.

(A-E) Whole cell current clamp recordings of identified CINs expressing tdTomato (n = 7 neurons) before (C) and after (D) bath application of CNO (clozapine-N-oxide, 10μM). A significant decrease in the neuron activity was observed (paired one side t-test: $t(6) = 3.677$, $P = 0.0104$).

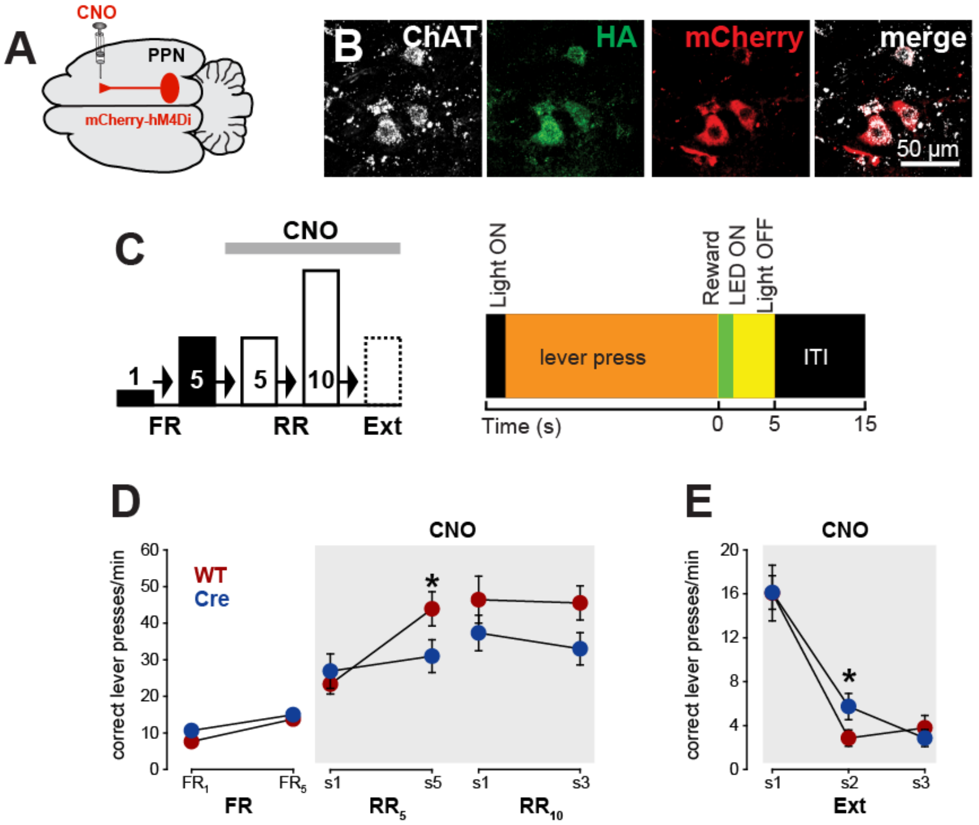

Supplementary Figure 9: Effects of chemogenetic inhibition in a random ratio lever press task. Related to Figures 6 and 7.

(A-B) ChAT::Cre+ rats (n = 14) were transduced with of AAV-DIO-hM4Di-HA-mCherry in the PPN and implanted with cannulas in the DLS to test the role of cholinergic neurons in a progressive ratio lever press task, a behavioral paradigm that has previously been shown to be affected following the excitotoxic lesion of the PPN (Wilson et al., 2019). Wild-type animals were injected with the same virus and implanted with cannulas.

(C) Rats were first trained on continous reinforcement (CRF) to reach a criterion of 80 correct presses in a 30 minutes schedule. Following acquisition of the CRF criterion, animals were switched to a random ratio schedule RR5 (5 days), RR10 (3 days) followed by 3 days of reward extinction. Animals received a local injection of CNO.

(D) No difference was observed on the first session of RR5 (RR5-1, CRE: 26.90 +/- 4.73, WT: 23.3 +/- 2.65) while at the last session of RR5 we observed an increase of lever presses following local administration of CNO (RR5-5: WT:43.92 +/- 4.69, ChAT: 30.99 +/- 4.5, mixed ANOVA group * day: $F(1,23)$ =28.23 Pday=0.0003; $F(1,23)$=2.26, Pgroup=0.1036, $F(1,23)$=13.55, Pinteraction=0.0042, Bonferroni posthoc: WT: P=0.0053, CRE: P=0.13). Following switch to RR10, we observed no significant difference between group on the first day (RR10-1: WT: 46.43+/-6.39, CRE; 37.34 +/- 4.83, P=0.3094) and the last day (RR10-3: WT: 45.55+/-4.65, CRE: 33.01+/-4.4, P=0.0842, n=7 rats). (E) During lever press extinction (3 consecutive days), a slower extinction in ChAT-Cre rats was observed (mixed ANOVA day*group: $F(2,35)$=86.51, Pday=0.00001, day 2: P=0.043, n=7 rats).

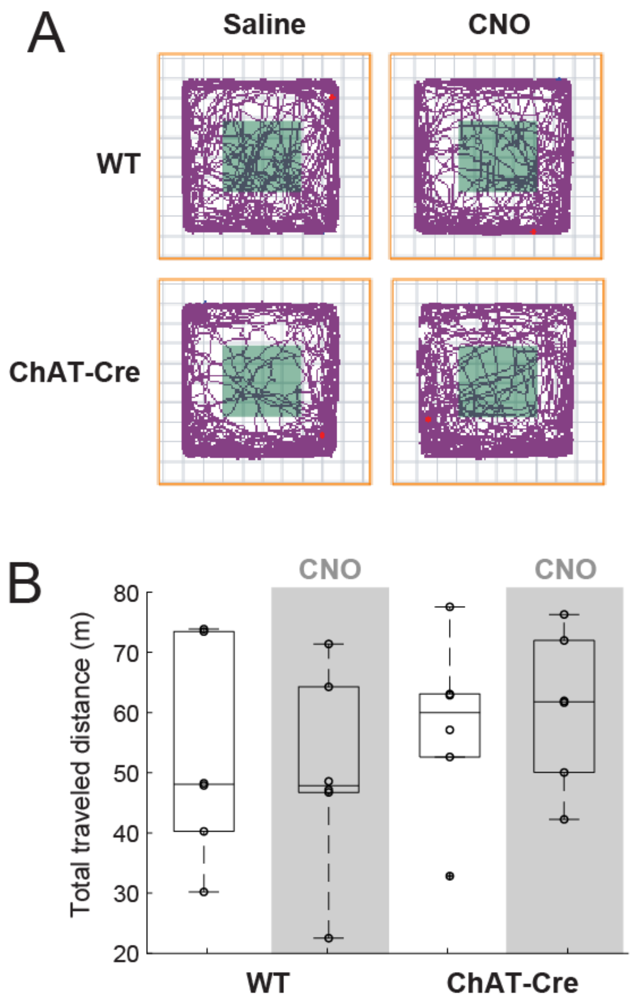

Supplementary Figure 10: Absence of motor effects during chemogenetic inhibition of PPN cholinergic neurons. Related to Figures 6 and 7.

Following the transduction of hM4Di in ChAT::Cre+ rats, transgenic and WT animals were analyzed during open field locomotion following the administration of CNO or saline. (A) Representative traces of total distance traveled in the open field during a 60 min session following the i.p. injection of CNO or saline. The total size of the arena is 1600 cm2. The center zone is marked with green. The total area of the center zone is 400 cm2. (B) No significant differences in total distance travelled were associated with the treatment or the phenotype (n= 7 rats). Box plots are represented using the mean, median, minimum and maximum value first quartile and third quartile.

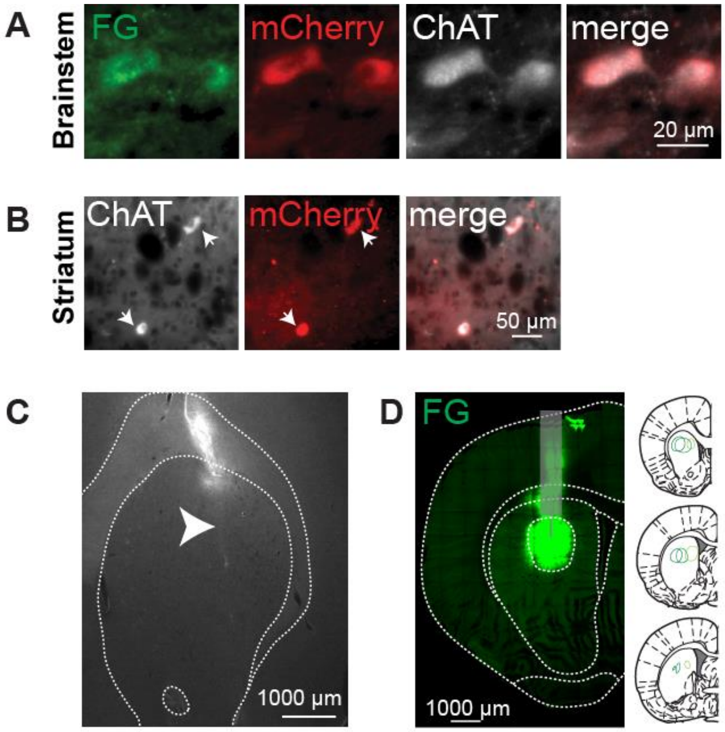

Supplementary Figure 11. Histology from behavioral experiments. Related to Figures 6 and 7.

(A) Cholinergic neurons in the midbrain expressing mCherry were retrogradely-labeled following injection of Fluorogold (FG) through the cannula in the striatum used for CNO delivery.

(B) Following injection of AAV-DIO-Hm4Di-HA-mCherry in the striatum, expression of mCherry was observed only in neurons positively labeled for ChAT. No retrograde mCherry labeling was observed in the midbrain.

(C) Location of the cannula in the striatum (DLS in this example) determined by FG.

(D) Diffusion of FG in the striatum. The volume of FG was the same as the volume of CNO to determine the extent of diffusion within the striatum (see text for details).

All experiments have been replicated at least 3 times.

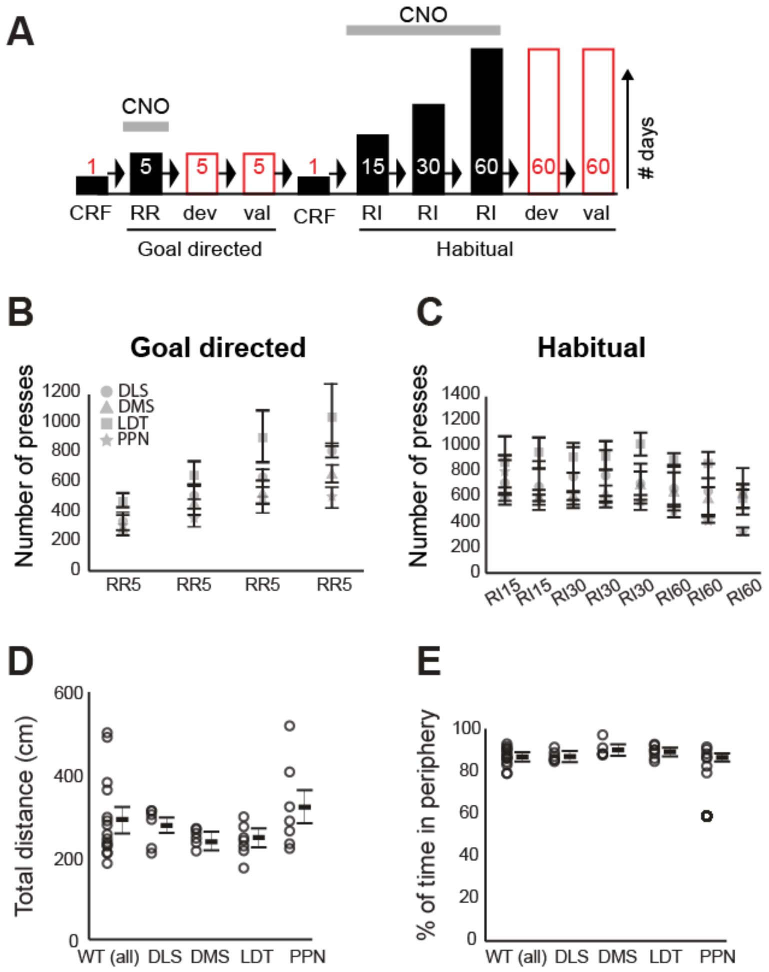

Supplementary Figure 12. Design of behavioral experiments. Related to Figure 6 and 7.

(A) Schematic of the experimental design illustrating the training and the timing of intracranial CNO administration. The height of the bars represents the number of experimental days.

(B-C) Acquisition of lever press in control (WT, n= 20 rats) rats under RR (B, two-way ANOVA group x day: $F_{interaction}(9,64)=0.63$, P=0.765) and RI (C, two-way ANOVA group x day: Finteraction(24,144)=0.48, P=0.981) contexts revealed no significant differences in the number of lever presses between groups.

(D-E) Effect of virus injections and CNO on locomotion (D; two-way ANOVA group x drug: F_{group} (3,35)=1.69, *P*=0.1924 ; $F_{drugs}(1,35)=1.86$, *P*=0.1840; $F_{interaction}(3,35)=0.92$, *P*=0.4461) or anxiety related behavior (E; two-way ANOVA group x drugs condition: F_{group} (3,35)=1.06, *P*=0.3820; $F_{drugs}(1,35)=0.61$, *P*=0.4429; $F_{interaction}(3,35)=2.48$, *P*=0.0815) revealed no significant effects across groups (n=50 rats).

Individual data points and mean ± SEM are shown.

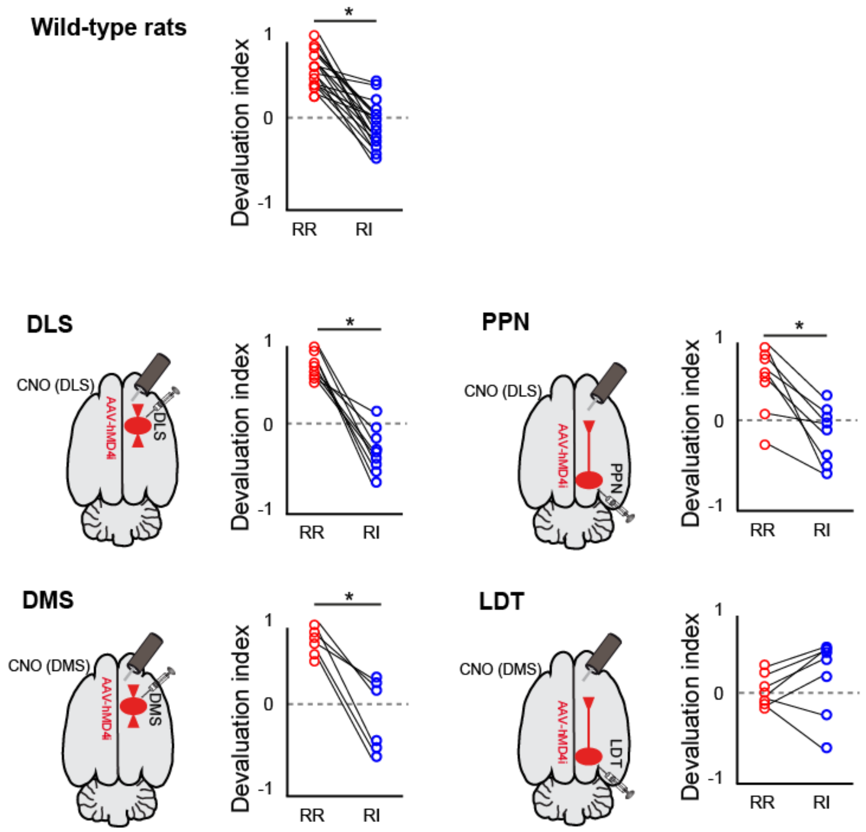

Supplementary Figure 13. Devaluation index of the number of presses. Related to Figures 6 and 7.

The difference in the proportion of responses between valued and devalued sessions was calculated from the normalized number of presses and obtained as an index. This difference (devaluation index) was significantly different in all groups except the LDT group (paired 2-tailed t-test: DLS t(8) = 7.704, P = 0.000057; PPN t(7) = 2.739, P = 0.029; DMS t(5) = 4.299, P = 0.008, n= 50 rats).
