# Supplementary material for: Cholinergic midbrain afferents modulate striatal circuits and shape encoding of action strategies
Source: Nat Commun. 2020 Apr 8;11:1739. doi: 10.1038/s41467-020-15514-3 (PMC7142106; doi:10.1038/s41467-020-15514-3)
Supplement: Supplementary file 3 — Reporting Summary [file 41467_2020_15514_MOESM3_ESM.pdf]

nature research

Corresponding author(s): Juan-Mena Segovia
Last updated by author(s): Mar 3, 2020

Reporting Summary

Nature Research wishes to improve the reproducibility of the work that we publish. This form provides structure for consistency and transparency in reporting. For further information on Nature Research policies, see Authors & Referees and the Editorial Policy Checklist .

Statistics

For all statistical analyses, confirm that the following items are present in the figure legend, table legend, main text, or Methods section.

n/a	Confirmed	
☐	☒	The exact sample size (n) for each experimental group/condition, given as a discrete number and unit of measurement
☐	☒	A statement on whether measurements were taken from distinct samples or whether the same sample was measured repeatedly
☐	☒	The statistical test(s) used AND whether they are one- or two-sided<br>*Only common tests should be described solely by name; describe more complex techniques in the Methods section.*
☐	☒	A description of all covariates tested
☐	☒	A description of any assumptions or corrections, such as tests of normality and adjustment for multiple comparisons
☐	☒	A full description of the statistical parameters including central tendency (e.g. means) or other basic estimates (e.g. regression coefficient) AND variation (e.g. standard deviation) or associated estimates of uncertainty (e.g. confidence intervals)
☐	☒	For null hypothesis testing, the test statistic (e.g. F, t, r) with confidence intervals, effect sizes, degrees of freedom and P value noted<br>*Give P values as exact values whenever suitable.*
☒	☐	For Bayesian analysis, information on the choice of priors and Markov chain Monte Carlo settings
☒	☐	For hierarchical and complex designs, identification of the appropriate level for tests and full reporting of outcomes
☒	☐	Estimates of effect sizes (e.g. Cohen's d, Pearson's r), indicating how they were calculated

Our web collection on statistics for biologists contains articles on many of the points above.

Software and code

Policy information about availability of computer code

Data collection	Spike2 7.0, Matlab 2018b , Arduino 1.8.1, ImageJ 1.41
Data analysis	Spike2 7.0, Matlab 2018b, ImageJ 1.41, SPSS 25.0

For manuscripts utilizing custom algorithms or software that are central to the research but not yet described in published literature, software must be made available to editors/reviewers. We strongly encourage code deposition in a community repository (e.g. GitHub). See the Nature Research guidelines for submitting code & software for further information.

Data

Policy information about availability of data

All manuscripts must include a data availability statement. This statement should provide the following information, where applicable:

- Accession codes, unique identifiers, or web links for publicly available datasets
- A list of figures that have associated raw data
- A description of any restrictions on data availability

In vivo electrophysiology data, in vitro electrophysiology and behavior data are available under reasonable request to the corresponding author.

Field-specific reporting

Please select the one below that is the best fit for your research. If you are not sure, read the appropriate sections before making your selection.

☒ Life sciences ☐ Behavioural & social sciences ☐ Ecological, evolutionary & environmental sciences

For a reference copy of the document with all sections, see nature.com/documents/nr-reporting-summary-flat.pdf

Life sciences study design

All studies must disclose on these points even when the disclosure is negative.

Sample size	No statistical methods were used to predetermine sample size, but all sample sizes were similar to previously reported in vivo electrophysiological (Dautan et al., 2016), anatomical (Dautan et al., 2014, 2016) or behavioral studies (Gremel and Costa, 2016; Gremel et al., 2013).
Data exclusions	Animals with injections or implantation sites out of target were excluded using pre-established criteria.
Replication	Data were collected from different sample and groups. For behavioral experiments, WT animals were injected with same virus as ChAT-cre animals, and considered as controls. For in vivo electrophysiology, control animals were injected with a reporter virus and processed similarly as experimental animals. All experiments, including in vivo and ex vivo electrophysiology, behavioral experiments and anatomical tracing were replicated a minimum of 3 times.
Randomization	In most of the experiments, animals were randomly assigned to the control and experimental groups. For the behavioral experiments, drugs, testing boxes, testing order and devaluation order were randomized using unbiased methods, and animals were allocated to their groups based on their genotype (WT or ChAT-cre).
Blinding	No blinding was used. All animals were assigned to their respective groups before the initiation of the experiments based on their virus injection or genotype.

Reporting for specific materials, systems and methods

We require information from authors about some types of materials, experimental systems and methods used in many studies. Here, indicate whether each material, system or method listed is relevant to your study. If you are not sure if a list item applies to your research, read the appropriate section before selecting a response.

Materials & experimental systems

n/a	Involved in the study	
☐	☒	Antibodies
☒	☐	Eukaryotic cell lines
☒	☐	Palaeontology
☐	☒	Animals and other organisms
☒	☐	Human research participants
☒	☐	Clinical data

Methods

n/a	Involved in the study	
☒	☐	ChIP-seq
☒	☐	Flow cytometry
☒	☐	MRI-based neuroimaging

Antibodies

Antibodies used

Target / Raised in / Cat. number / Dilution / Company

Primary antibodies:
ChAT Goat AB144P 1/500 Millipore Sigma
GFP Rabbit A21311 1/1000 Invitrogen
PV Guinea Pig 195004 1/500 Synaptic Systems
Ctip2 Rabbit AB28448 1/500 Abcam
HA Rabbit 3724 1/1000 Cell Signaling
mCherry Rabbit AB167453 1/1000 Abcam
pser240-244 Rabbit 2215 1/250 Cell Signaling
GFP Rat IgG2a 04404 1/1000 Nacalai Tesque

Secondary antibodies:
anti-Goat CY5 Donkey 705-175-147 1/250 Jackson Immunoresearch
anti-Goat CY3 Donkey 705-165-147 1/500 Jackson Immunoresearch
anti-Rabbit 488 Donkey 711-545-152 1/500 Jackson Immunoresearch
anti-Rabbit CY3 Donkey 711-165-152 1/500 Jackson Immunoresearch
anti-Guinea pig 488 Donkey 706-545-148 1/500 Jackson Immunoresearch
anti-rat biotinylated Rabbit BA4000 1/500 Vector Labs
anti-goat IgG Donkey 705-005-147 Jackson Immunoresearch
PAP Goat 123-005-024 1/200 Jackson Immunoresearch

Validation

Primaries:
ChAT Goat AB144P: Affinity purified. Routinely evaluated by Western Blot on mouse brain lysates.

GFP Rabbit A21311: Polyclonal. Isolated direclty from the jellyfish Aequorea victoria. Used and validated by IHC, flow cytometry, Western Blot, Immunoprecipitation
PV guinea pig 195004: Recombinant protein corresponding to AA 1 to 110 from rat PV. Validated by IHC and Western Blot
Ctip2 Rabbit AB28448: Polyclonal affinity purified Synthetic peptide conjugated to KLH derived from within residues 850-950 of Human Ctip2. This antibody gave positive signal in the following human cell lysates: Jurkat and Jurkat nuclear.
HA Rabbit 3724 Monoclonal antibody: produced by immunizing animals with a synthetic peptide containing the influenza hemagglutinin epitope (YPYDVPDYA). Antibody validated by IHC, Western Blot and immunoprecipitation.
mCherry Rabbit AB167453: Polyclonal. Recombinant full length corresponding to mCherry. Positive control: HEK293 cells transefected with pFin-EF1-mCherry vector. Validated by IHC and Western Blot.
pser 240-244 Rabbit 2215: Polyclonal antibody purified by protein A and peptide affinity chromatography.
GFP Rat 04404: Clone GF090R. His-GFP full length fusion protein. Validated by IHC, Western Blot and ELISA.

Secondaries:
anti-Goat Cy5 and Cy3 Donkey, anti-rabbit 488 and Cy3 Donkey & anti-guinea pig 488 Donkey: These antibodies were purified from antisera by immunoaffinity chromatography using antigens coupled to agarose beads. Based on immunoelectrophoresis and/or ELISA the antibody reacts with whole molecule IgG.
anti-rat biotinylated BA4000: High affinity antibody. Purified by affinity chromatography. Cross-reactivities that are likely to interfere with specific labeling are removed by solid phase adsorption techniques.
anti-goat IgG 705-005-147: The antibody was purified from antisera by immunoaffinity chromatography using antigens coupled to agarose beads. Based on immunoelectrophoresis and/or ELISA the antibody reacts with whole molecule goat IgG.
goat-PAP 123-005-024: PAP soluble complexes are prepared by the method of Stenberger et al (J. Histochem. 1970, 18, 315). They consist of two anti-horseradish peroxidase antibodies in soluble complex with three molecules of horseradish peroxidase.

Animals and other organisms

Policy information about studies involving animals; ARRIVE guidelines recommended for reporting animal research

Laboratory animals	Male and female adult (250-450g, 2-8 months of age) Long Evans (LE) wild type and ChAT::cre+ rats were used for all anatomical (20 female/2 male), in vivo electrophysiological (27 female/39 male) and behavioral experiments (18 female/70 male).
Wild animals	No wild animals have been used for the study.
Field-collected samples	No field collected animals have been used for the study.
Ethics oversight	The Rutgers University Institutional Animal Care and Use Committee and in accordance with the NIH Guide to the Care and Use of Laboratory Animals, and the Animals (Scientific Procedures) Act, 1896 (UK), under the authority of Project License approved by the Home Office.

Note that full information on the approval of the study protocol must also be provided in the manuscript.
