# Supplementary material for: Cholinergic midbrain afferents modulate striatal circuits and shape encoding of action strategies
Source: Nat Commun. 2020 Apr 8;11:1739. doi: 10.1038/s41467-020-15514-3 (PMC7142106; doi:10.1038/s41467-020-15514-3)
Supplement: Supplementary file 2 — Peer Review [file 41467_2020_15514_MOESM2_ESM.pdf]

**Editorial Note:** This manuscript has been previously reviewed at another journal that is not operating a transparent peer review scheme. This document only contains reviewer comments and rebuttal letters for versions considered at *Nature Communications.*
